# Neutrophil extracellular traps have auto-catabolic activity and produce mononucleosome-associated circulating DNA

**DOI:** 10.1186/s13073-022-01125-8

**Published:** 2022-11-28

**Authors:** Ekaterina Pisareva, Lucia Mihalovičová, Brice Pastor, Andrei Kudriavtsev, Alexia Mirandola, Thibault Mazard, Stephanie Badiou, Ulrich Maus, Lena Ostermann, Julia Weinmann-Menke, Elmo W. I. Neuberger, Perikles Simon, Alain R. Thierry

**Affiliations:** 1 IRCM, Institut de Recherche en Cancérologie de Montpellier, INSERM U1194, Université de Montpellier, Institut Régional du Cancer de Montpellier, 34298 Montpellier, France; 2 Institute of Molecular Biomedicine, Faculty of Medicine, Comenius University, Sasinkova 4, 811 08 Bratislava, Slovakia; 3 Department of Medical Oncology, Montpellier Cancer Institute (ICM), Montpellier, France; 4 Laboratoire de Biochimie Et Hormonologie, PhyMedExp, Université de Montpellier, INSERM, CNRS, CHU de Montpellier, Montpellier, France; 5 Division of Experimental Pneumology, Hannover Medical School, and German Center for Lung Research, Partner Site BREATH (Biomedical Research in Endstage and Obstructive Lung Disease), 30625 Hannover, Germany; 6 Department of Rheumatology and Nephrology, University Medical Center Mainz, Langenbeckstr. 1, 55101 Mainz, Germany; 7 Department of Sports Medicine, University of Mainz, Albert-Schweitzer Str. 22, 55128 Mainz, Germany; 8 Montpellier Cancer Institute (ICM), Montpellier, France

**Keywords:** Circulating DNA, Elastase, Myeloperoxidase, Neutrophil, NET, Nucleosome

## Abstract

**Background:**

As circulating DNA (cirDNA) is mainly detected as mononucleosome-associated circulating DNA (mono-N cirDNA) in blood, apoptosis has until now been considered as the main source of cirDNA. The mechanism of cirDNA release into the circulation, however, is still not fully understood. This work addresses that knowledge gap, working from the postulate that neutrophil extracellular traps (NET) may be a source of cirDNA, and by investigating whether NET may directly produce mono-N cirDNA.

**Methods:**

We studied (1) the in vitro kinetics of cell derived genomic high molecular weight (gHMW) DNA degradation in serum; (2) the production of extracellular DNA and NET markers such as neutrophil elastase (NE) and myeloperoxidase (MPO) by ex vivo activated neutrophils; and (3) the in vitro NET degradation in serum; for this, we exploited the synergistic analytical information provided by specifically quantifying DNA by qPCR, and used shallow WGS and capillary electrophoresis to perform fragment size analysis. We also performed an in vivo study in knockout mice, and an in vitro study of gHMW DNA degradation, to elucidate the role of NE and MPO in effecting DNA degradation and fragmentation. We then compared the NET-associated markers and fragmentation size profiles of cirDNA in plasma obtained from patients with inflammatory diseases found to be associated with NET formation and high levels of cirDNA (COVID-19, *N* = 28; systemic lupus erythematosus, *N* = 10; metastatic colorectal cancer, *N* = 10; and from healthy individuals, *N* = 114).

**Results:**

Our studies reveal that gHMW DNA degradation in serum results in the accumulation of mono-N DNA (81.3% of the remaining DNA following 24 h incubation in serum corresponded to mono-N DNA); “ex vivo” NET formation, as demonstrated by a concurrent 5-, 5-, and 35-fold increase of NE, MPO, and cell-free DNA (cfDNA) concentration in PMA-activated neutrophil culture supernatant, leads to the release of high molecular weight DNA that degrades down to mono-N in serum; NET mainly in the form of gHMW DNA generate mono-N cirDNA (2 and 41% of the remaining DNA after 2 h in serum corresponded to 1–10 kbp fragments and mono-N, respectively) independent of any cellular process when degraded in serum; NE and MPO may contribute synergistically to NET autocatabolism, resulting in a 25-fold decrease in total DNA concentration and a DNA fragment size profile similar to that observed from cirDNA following 8 h incubation with both NE and MPO; the cirDNA size profile of NE KO mice significantly differed from that of the WT, suggesting NE involvement in DNA degradation; and a significant increase in the levels of NE, MPO, and cirDNA was detected in plasma samples from lupus, COVID-19, and mCRC, showing a high correlation with these inflammatory diseases, while no correlation of NE and MPO with cirDNA was found in HI.

**Conclusions:**

Our work describes the mechanisms by which NET and cirDNA are linked. In doing so, we demonstrate that NET are a major source of mono-N cirDNA independent of apoptosis and establish a new paradigm of the mechanisms of cirDNA release in normal and pathological conditions. We also demonstrate a link between immune response and cirDNA.

**Supplementary Information:**

The online version contains supplementary material available at 10.1186/s13073-022-01125-8.

## Background

CirDNA [1] has gained considerable attention in recent decades because of its huge potential for clinical diagnostics. It is already being used for prenatal diagnosis, liquid biopsy in oncology, and other medical applications such as transplantation or non-invasive prenatal testing [2, 3]. Further optimization of cirDNA diagnostics would open up significant perspectives for personalized medicine, but that requires an accurate understanding of the fundamental mechanisms underlying the release of DNA into the circulation, of their fragmentation, and of their cellular/tissue of origin.

It was initially thought that cirDNA could be up to 40 kb in size, but was principally 180 bp (or multiples thereof), which corresponds to the size of DNA packed in a mononucleosome [4, 5]. Observations of mono- and oligonucleosomes led to the view that the major mechanism of cirDNA release is apoptosis [3, 5, 6]. Alternatively, necrosis was reported as playing a major role in response to ionizing radiation [7]. It has also been shown that DNases are the main actors of DNA degradation [8], and the particular endonucleases to which that degradation has been attributed include caspase-activated DNAse (CAD), DNase 1L3, Endonuclease G (Endo G), and DNase I [9–14]. These endonucleases show high DNA degradation activity in blood, which is considered a normal physiological phenomenon whose purpose is to maintain homeostasis in the body and to protect against viruses [15, 16]. However, the kinetics of long DNA fragmentation by serum nucleases and its role in the appearance of chromatosome-associated cirDNA fragments in the blood have yet to be fully elucidated.

NET were discovered in 2004 [17] and have been presented as a potential bactericidal killing mechanism. Their mechanism has been described as involving the activation of neutrophils to release their DNA, which is laden with powerful enzymes (such as neutrophil elastase and myeloperoxidase), that traps and kills microbes [18]. Interestingly, NET are found in the same pathological conditions where high concentrations of cirDNA have been reported, such as autoimmune diseases [19], inflammatory diseases [20], sepsis [21], thrombotic illnesses [22], and cancer [23–25]. However, despite the clinical potential inherent in such observations, no report exists which delineates the mechanistic process by which genomic DNA from NET is degraded into circulating mononucleosomes. Our studies now address that. Specifically, we believe that chromatin fragments, oligonucleosomes, and nucleosomes can be liberated by the process of NET degradation which occurs during inflammation and that these contribute to the pool of cirDNA and histones in blood [25]. We have already demonstrated the association of NET formation with cirDNA production in metastatic colorectal cancer [26], and we also postulate that NET have a key role in COVID-19 pathogenesis [25].

The fragmentation of cirDNA and NET-associated biomarkers is of particular interest in identifying the cirDNA’s cell/tissue-of-origin, and as such could have numerous clinical applications. It has also been shown that upon activation, neutrophil elastase (NE) escapes from azurophilic granules and translocates to the nucleus, where it partially degrades specific histones, promoting chromatin decondensation. Subsequently, myeloperoxidase (MPO) synergizes with NE in driving chromatin decondensation independent of its enzymatic activity [27]. These data led us to suggest that NET-associated enzymes could modify the cirDNA structure and, thus, change its fragment size distribution in patients with NET-associated disorders. We have demonstrated the particular fragmentation of cirDNA in the plasma of cancer patients and were the first to postulate that cirDNA fragment size profile (fragmentomics, [28]) could be used to distinguish the cirDNA of cancer and healthy subjects [29, 30]. Our recent fragmentomics studies based on shallow whole genome sequencing (sWGS) revealed the detailed cirDNA size profiles associated with chromatosome structures in the plasma of healthy individuals (HI) and cancer patients [31]. Thus, cirDNA fragmentation pattern and NET-associated biomarkers are being studied as a possible means of stratifying individuals [29–35].

**Fig. 1:**
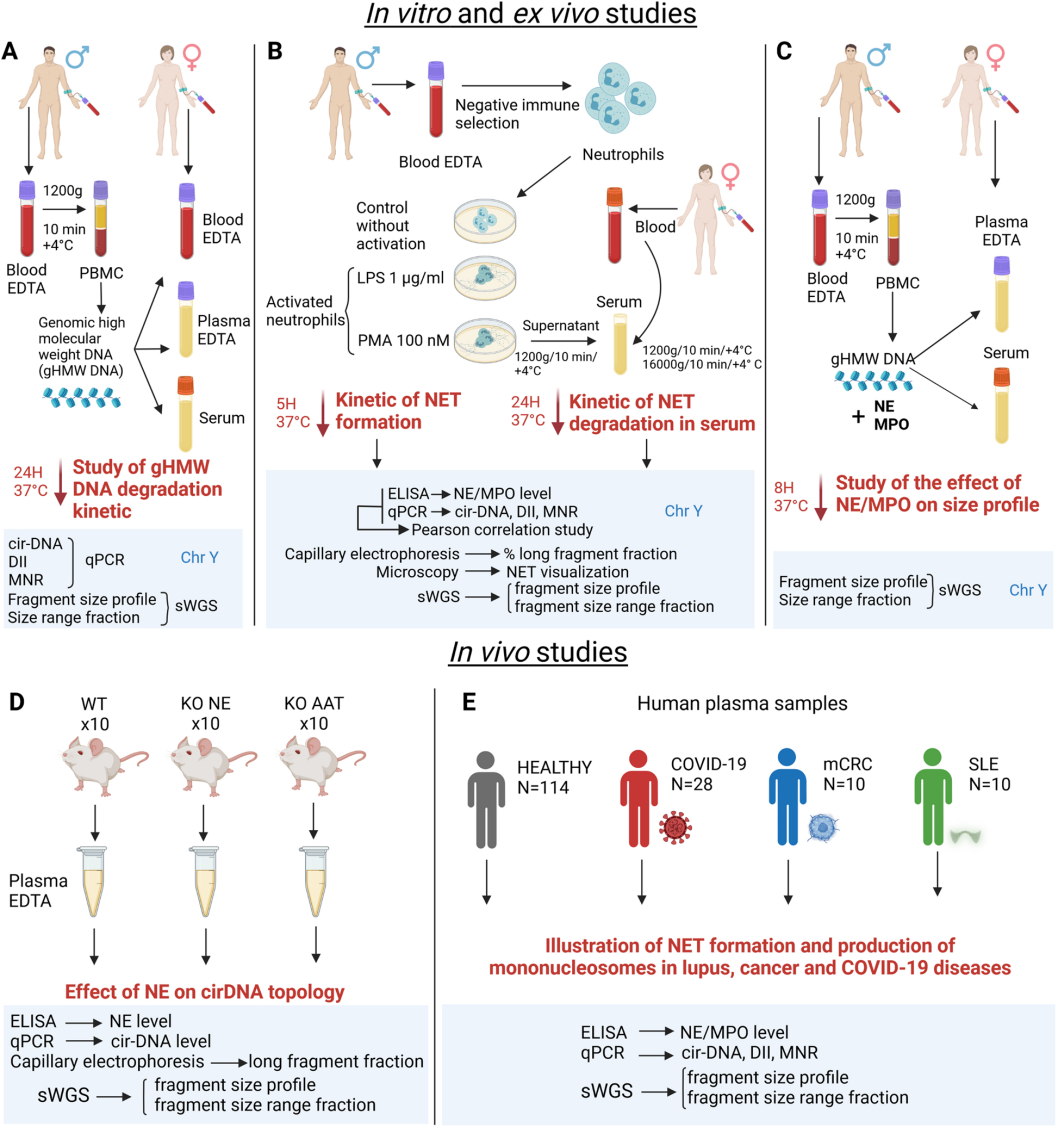
Schematic overview of the in vitro, ex vivo, and in vivo studies of cirDNA production by NET.** A **In vitro study of gHMW DNA degradation in blood fluids using Y chromosome analysis. **B **Ex vivo study of NET production and degradation using Y chromosome analysis. **C **In vitro study of the effect of NE/MPO on cfDNA size profile using Y chromosome analysis. **D **In vivo study of the effect of NE on cirDNA topology. **E **In vivo study of NET-associated disorders

In the present study (Fig. 1), we used the synergistic analytical information provided by qPCR, sWGS, and capillary electrophoresis to unequivocally observe the following: the kinetics of genomic DNA degradation by serum nucleases in vitro; the production of extracellular DNA (or cfDNA) by ex vivo activated neutrophils; the in vitro NET degradation by serum nucleases. This enabled us to precisely measure cirDNA size over a wide range of lengths, thus obtaining information about DNA structures which can be detected during the processes of NET production and degradation. We also performed an in vivo study in knockout mice, and an in vitro study of genomic high molecular weight (gHMW) DNA degradation, in order to elucidate the role of NE and MPO in cirDNA fragmentation. We then compared the NET-associated markers and fragmentation size profiles of cirDNA in plasma obtained from patients with COVID-19, systemic lupus erythematosus (SLE) patients, metastatic colorectal cancer (mCRC) patients, and healthy individuals (HI).

## Methods

### Patients and healthy individuals

Plasma from blood samples collected from 10 consecutive mCRC patients, obtained via the screening procedure of the ongoing multicentric UCGI 28 PANIRINOX study (NCT02980510/EudraCT n°2016–001,490-33), were isolated by the IRCM in the course of this study (August-December 2020). Plasma samples from 28 patients with severe COVID-19 (at the entrance of Intensive Care Unit) were provided by the CHU hospital of Montpellier (Centre Hospitalier Universitaire de Montpellier, France); approval number assigned by the IRB: IRB-MTP_2021_01_202100715 [36]. One to 2 mL of blood were consecutively collected from COVID patients between March and May 2021 (Delta SARS-Cov2 infection). Plasma samples from 10 female patients with systemic lupus erythematosus (SLE) were provided by Dr. P. Simon from the Department of Sports Medicine, Prevention and Rehabilitation of Johannes Gutenberg University (Mainz, Germany). These samples originate from a clinical study that was approved by the Human Ethics Committee of Rhineland-Palatinate, Germany (number 2018–13,039) and conformed to the standards of the Declaration of Helsinki of the World Medical Association and was registered under ClinicalTrials.gov Identifier: NCT03942718. We also analyzed 114 healthy individuals (HI, 59 men and 55 women) from the Etablissement Français du Sang (EFS), which is Montpellier’s blood transfusion center under the EFS-PM N° 21PLER2015-0013 agreement number. These samples were initially screened (virology, serology, immunology, blood numeration) and ruled out wherever any abnormality was detected. Note, data presented in this study are not related to the outcome of the above clinical trials.

### Animals

Animal experiments involving WT, NE, and AAT KO mice corresponded to the European guideline 2010/63/EU and were authorized by the Lower Saxony State Office for Consumer Protection and Food Safety, Wardenburg, Germany (permission 18/2894). All mice were maintained under specific-pathogen free (SPF) conditions at ambient temperature (22 °C) using 14/10 h bright/dark light exposition. Animals were fed with standard laboratory mouse diet (Altromin 1324 TPF, Lage, Germany) and water ad libitum. For euthanasia, mice were exposed to isoflurane until asphyxia followed by immediate drawing of blood from the Vena cava to ensure death of mice. Treatment of mice conformed to the standards of the German Animal Welfare law and the Declaration of Helsinki.

### Extracellular DNA nomenclature

We recently published the first article proposing a standard nomenclature to be used in the cirDNA field, to homogenize the various terminologies currently in use [1]. Thus, we clearly differentiate cell-free DNA (cfDNA), which can be found in various biological sources (cell culture supernatant, blood, urine, pap smears, LCR …), from circulating DNA, which is found only in blood or lymph nodes. While both groups share numerous characteristics, their physiological localization results in clear differences, as shown for instance by their fragment size profile (i.e., blood vs urine) [1]. In this study, we clearly differentiate cfDNA, detected in the gHMW DNA or NET in vitro degradation experiments, from cirDNA, detected in plasma from human or mice subjects.

### gHMW DNA preparation

A blood sample from a male healthy individual was collected in a 10-ml EDTA tube. After centrifugation of the blood at 1200* g* for 10 min at + 4 °C, the PBMC layer was collected into a separate tube. The PBMCs were washed twice with PBS and pelleted by centrifugation at 800* g*. The supernatant was discarded, and the cell pellet resuspended in 1 ml of PBS with the addition of a protease inhibitor cocktail (cOmplete™ Protease Inhibitor Cocktail, Roche) in order to protect histones from degradation by proteases during the next step of cell lysis. The cells were lysed by 10 freeze–thaw cycles. The lysate was centrifuged at 800* g* for 10 min at + 4 °C, in order to precipitate the non-lysed cells. The supernatant of this cell extract containing a high molecular weight genomic DNA (gHMW DNA, chromatin) was collected and used for the following experiments on degradation of DNA in serum and plasma. The 25 µL aliquot of the cell extract was used for quality and quantity control of chromatin in the cell extract. The DNA was extracted using a QIAamp DNA blood mini kit (Qiagen). Its quantity and integrity were analyzed by (1) qPCR, using the two pairs of primers for amplification of the short and long fragments (67 bp and 320 target on *KRAS* gene); and by (2) capillary electrophoresis on Fragment Analyzer (Agilent, HS Large Fragment Kit DNF-493). The quality control of gHMW DNA before degradation showed a DNA concentration of 10 ng/µL, and DNA fragments ranging from 2 to 40 kb, peaking at 12 kb (Additional file 1: Fig. S1).

### Plasma/serum isolation

Samples were handled according to preanalytical guidelines previously established by our group [37]. Blood samples from HI were collected in 10-ml EDTA tubes (K2E, REF 367,525, BD Vacutainer) for plasma or in 4-ml CAT tubes (Clot Activator Tube, REF 369,032, BD Vacutainer) for serum. Serum was used only for the experiments involving gHMW DNA or NET degradation. Blood from mCRC patients (*n* = 10) was collected in STRECK tubes (Cell-Free DNA BCT®) and was sent within 24 h of collection at room temperature from the recruiting institutions to our laboratory (IRCM, Institut de Recherche en Cancérologie de Montpellier, U1194 INSERM). The blood was then centrifuged at 1200* g* at 4 °C for 10 min. The supernatants were isolated in sterile 1.5-mL Eppendorf tubes and centrifuged at 16,000* g* at 4 °C for 10 min [29]. Isolation of plasma from COVID-19 and lupus individuals was performed under the same protocol, but using EDTA tubes. Afterwards, the plasma or serum was either immediately used for the experiment on gHMW DNA or NET degradation, or was stored at − 20 °C until cirDNA extraction or ELISA analysis.

### Design of experiment for the study of gHMW DNA and NET degradation

The study of gHMW DNA and NET degradation was done by incubation of the chromatin from PBMC lysate in blood fluids. We used gHMW DNA from a male donor and blood/plasma/serum from a female donor, to further analyze Y chromosome fragments that belong exclusively to the gHMW DNA under study, and which obviously are absent in the plasma of the female donor. The size profile of the degraded Y chromosome was studied by qPCR and shallow WGS.

### Degradation of gHMW DNA in blood fluids

The 20 µl of chromatin equivalent to 200 ng of genomic DNA was added to 5 aliquots of 500 µL of blood EDTA, plasma EDTA, or serum. These samples were incubated at 37 °C for 0 min, 30 min, 2 h, 8 h, and 24 h. After incubation, the plasma was isolated from blood EDTA as described above. Then, cfDNA was extracted from 0.5 ml of plasma or serum using the QIAmp DNA Mini Blood kit (Qiagen, CA), according to the “Blood and body fluid protocol” and our own detailed protocol [38]. CfDNA samples were kept at 20 °C until use. Due to EDTA-associated calcium chelation inhibitory effect on nucleases, comparison of plasma vs serum incubation may help in estimating nuclease role in degrading gHMW DNA.

### Degradation of gHMW DNA in blood fluids in presence of NE and MPO

The 50 µl of chromatin equivalent to 500 ng of genomic DNA was added to 1 mL of plasma EDTA or serum. Then, 200 ng of NE (Sigma, 324,681) or 200 ng MPO (Sigma, 475,911) or both was added to the samples. Control samples were incubated under the same conditions, but without the addition of NE or MPO. These samples were incubated at 37 °C for 2 and 8 h. Then, cfDNA was extracted from plasma or serum using the QIAmp DNA Mini Blood kit (Qiagen, CA), according to the “Blood and body fluid protocol” and our own detailed protocol [38]. CfDNA samples were kept at 20 °C until use.

By subtracting values obtained in medium with NE/MPO from those obtained in the controls, we are able to estimate the DNA degradation rate in various conditions. Based on the strong inhibitory effect of EDTA on nuclease activity (95% and ~ 85% of DNA remain following 2 and 8 h, respectively), the overall, independent nuclease activity in serum is estimated by subtracting the DNA concentration in control serum from that found in control plasma over time. Assuming that overall DNA degradation in serum is due to the combined activities of nuclease and of NE/MPO, we estimated the degradation rate deriving specifically from NE, MPO, and NE plus MPO by subtracting the DNA concentration in serum to which NE and/or MPO has been added from that found in control serum over time. Overall degradation rates are expressed as ng DNA/mL/minutes and cannot be compared to an enzymatic activity.

### NET induction

For neutrophil preparation, blood from a healthy male volunteer was collected into two 10-ml EDTA tubes using standard phlebotomy techniques. Neutrophils were isolated from 20 ml of blood with EasySep™ Human Neutrophil Isolation Kit (STEMCELL Technologies, British Columbia, Canada, 19,666) according to the manufacturer’s instructions. The neutrophils were quantified using Trypan Blue staining on Countess II automated cell counter. Cell suspension was diluted to the cell concentration 5.6 × 10^5^ cells/ml with the RPMI medium containing 10% of serum from the donor of neutrophils. The neutrophils were seeded (5.6 × 10^5^ cells/ml; 1.1 million cells per well) in the 6-well plates (Falcon, REF 353,046). The neutrophils were incubated at 37 °C for 30 min, 1 h, 2 h, 4 h, and 5 h under the following conditions: control (non-stimulated neutrophils), neutrophils stimulated by PMA (PHORBOL 12-MYRISTATE 13-ACETATE, Sigma, P8139-1MG) in concentration 100 nM, and neutrophils stimulated by LPS (LIPOPOLYSACCHARIDES FROM ESCHERICHIA COLI, Sigma, L4391-1MG) in concentration 1 µg/ml. All experiments were performed in duplicate. After incubation, cell media was aspirated and centrifuged at 1200* g* for 10 min at + 4 °C in order to collect cell-free media.

### Degradation of NET in blood fluids

As part of our study, we also investigated the kinetics of in vitro degradation by serum nucleases of the NET produced in the previous part of the study where NET induction was studied. This allows a direct comparison of the levels of NET formation vs production. To do so, the previously obtained supernatants of PMA-stimulated (5 h) neutrophils from a male individual were incubated with a fresh female serum for various time periods. We also used the X/Y differentiation to distinguish NET-derived male DNA from female cirDNA derived from serum. The qPCR analysis was performed targeting the *SRY* gene on the Y chromosome, and sWGS results took into consideration only reads aligning on the Y chromosome.

The 200 µl of supernatant of the PMA-stimulated neutrophils (5 h) equivalent to 50 ng of genomic DNA was added to aliquots of 500 µL of plasma or serum. These samples were incubated at 37 °C for 5 min, 10 min, 20 min, 30 min, 2 h, 8 h, and 24 h for NET degradation in serum, with 24 h incubation in plasma used as a control. After incubation, cfDNA was extracted from 0.5 ml of plasma/serum using the QIAmp DNA Mini Blood kit (Qiagen, CA), performed according to the “Blood and body fluid protocol” and our own detailed protocol [38]. CfDNA samples were kept at − 20 °C until use.

### DNA extraction from plasma

CirDNA from plasma, serum or cell medium was extracted using the QIAmp DNA Mini Blood kit (Qiagen), used according to the “Blood and body fluid protocol,” using 80 µl of elution volume. DNA extracts were kept at − 20 °C until used.

### Quantification of DNA by qPCR

The concentration of cf- or cirDNA was assessed using an integrated PCR system that targeted sequences within the same region. The designed amplification sizes were 73 and 246 bp targeted *SRY* gene on the Y chromosome for cfDNA quantification; and 67 and 320 bp targeted *KRAS* gene for cirDNA quantification. This primer construction enabled the calculation of a DNA integrity index (DII) by calculating the ratio between the concentrations obtained with the primer pairs, by targeting both a long (246 or 320 bp) and a short (73 or 67 bp) sequence. Targeting long sequences (over 250 bp) are unable to amplify cirDNA fragments packaged in mononucleosome, while targeting short sequences (60–80 bp) amplifies almost all DNA fragments, including those associated with mononucleosomes (99% of cirDNA fragments from HI are detected by targeting a 60-bp sequence [31]). qPCR amplifications were carried out at least in duplicate in a 25 µL reaction volume on a CFX96 instrument using the CFX manager software (Bio-Rad). Each PCR reaction mixture was composed of 12.5 µl PCR mix (Bio-Rad Sso advanced mix SYBR Green), 2.5 µl of each amplification primer (0.3 pmol/ml, final concentration), 2.5 µl PCR-analyzed water, and 5 µl DNA extract. Thermal cycling consisted of three repeated steps: a 3 min Hot-start Polymerase activation and denaturation step at 95 °C, followed by 40 repeated cycles at 95 °C for 10 s, and then at 60 °C for 30 s. Melting curves were obtained by increasing the temperature from 55 to 90 °C, with a plate reading every 0.2 °C. The concentration was calculated from Cq detected by qPCR, and also a control standard curve on genomic DNA of known concentration and copy number. Serial dilutions of genomic DNA from human placenta (G1471, Promega) were used as a standard for quantification and their concentration.

### DNA analysis by capillary electrophoresis

The analysis of DNA by capillary electrophoresis was done using the Fragment Analyzer 5200 (Agilent) and DNF-493 kit, according to the manufacturer’s instructions.

### Library preparation for shallow WGS

In this study, we analyzed DNA size profiles using shallow WGS, which allows the accurate estimation of the number of fragments of a certain length with a resolution of 1 bp. DSP libraries were prepared with the NEBNext® Ultra™ II kit. For library preparation, a minimum of 1 ng of cf- or cirDNA was engaged without fragmentation, and recommendations from kit providers were followed. Briefly, for DSP with the NEB kit, Illumina paired-end adaptor oligonucleotides were ligated on repaired A-tailed fragments, then SPRI purified and enriched by 11 PCR cycles with UDI primers indexing, and then SPRI purified again. The SPRI purification was adjusted to keep the small fragments around 70b of insert. Finally, the libraries to be sequenced were then precisely quantified by qPCR, to ensure that the appropriate quantity was loaded on to the Illumina sequencer, in order to obtain a minimum of 1.5 million of clusters.

### Analysis of cirDNA by shallow WGS

All libraries were sequenced on MiSeq 500 or NovaSeq (Illumina) as Paired-end 100 reads. Image analysis and base calling was performed using Illumina Real Time Analysis with default parameters. The paired-end reads (PE) of individualy barcoded samples were trimmed with Cutadapt v1.10 to remove the adapters and discard trimmed reads shorter than 20 bp. Trimmed fastq files were aligned to the human reference genome (GRCH38) using the MEM algorithm in the Burrows-Wheeler Aligner (BWA) v0.7.15. The insert sizes were then extracted from the aligned bam files with the TLEN column for all pairs of reads having an insert size between 0 and 1000 bp. Supplementary alignments, PCR duplicates, and reads with MapQ < 20 were filtered out from the analysis. Summary statistics of sWGS data is presented in Additional file 1:Table S1. Sequencing data are published in Zenodo repository [39].

### Human myeloperoxidase (MPO) and neutrophil elastase (NE) quantifications

MPO and NE concentrations were measured using enzyme-linked immunosorbent assay (ELISA), according to the manufacturer’s standard protocol (Duoset R&D Systems, DY008, DY3174, and DY9167-05). Briefly, captured antibodies were diluted at the working concentrations in the Reagent Diluent (RD) provided in ancillary reagent kits (DY008), and coated overnight at room temperature (RT) on 96-well microplates with 100 μL per well. Then, captured antibodies were removed from the microplates, and wells were washed three times with 300 μL of wash buffer (WB). Microplates were blocked at RT for 2 h by adding 300 μL of RD to each well. RD were removed from the microplates, and wells were washed three times with 300 μL of WB. Then, 100 μL of negative controls, standards, and plasma samples (diluted 1/10) were added to the appropriate wells for 1 h at RT. Samples, controls, and standards were removed from the microplates, and wells were washed three times with 300 μL of WB. Detection antibodies were diluted at the working concentrations in the RD, then added by 100 μL per well, for 1 h at RT. Detection antibodies were removed from the microplates, and wells were washed three times with 300 μL of WB. Then, 100 μL of Streptavidin-HRP was added to each well and microplates were incubated at RT for 30 min. The wash was repeated three times. Finally, 100 μL per well of substrate solution was added and incubated for 15 min, and the optical density (OD) of each well was read immediately at 450 nm with the PHERAstar FS instrument using the PHERAstar control software.

### Statistical analysis

Statistical analysis was performed using the GraphPad Prism V6.01 software. Correlation analysis was performed using the Pearson test. The Mann–Whitney test was used to compare means. A probability of less than 0.05 was considered to be statistically significant; *p < 0.05, **p < 0.01; ***p < 0.001; ****p < 0.0001.

## Results

### Kinetics of gHMW DNA degradation in blood fluids

**Fig. 2:**
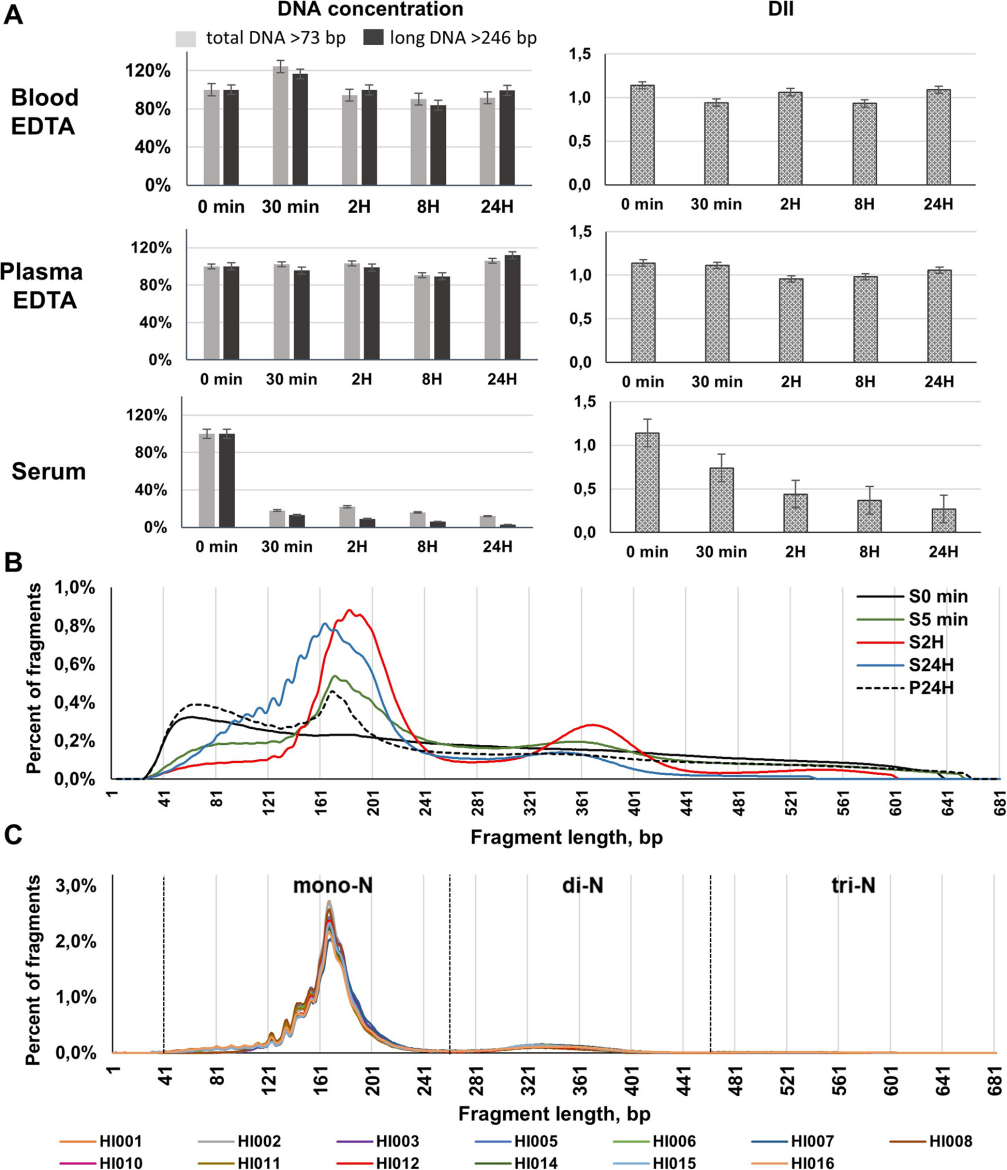
Kinetics of gHMW DNA degradation in blood fluids.** A** The kinetics of total and long DNA concentrations, as determined by qPCR, presented as a fraction of the initial concentration and DII (DNA integrity index) following gHMW DNA incubation up to 24 h at 37 °C in blood EDTA, plasma EDTA, and serum. Data are represented as mean ± SEM. **B** Size profiles of gHMW DNA samples before incubation in blood fluids (S0) and after incubation at 37 °C in plasma for 24 h (P24H) or in serum for 5 min (S5 min), 2 h (S2H), or 24 h (S24H) as determined by sWGS. **C** CirDNA fragment size profile of the thirteen healthy subjects, as determined by sWGS. Examination of gHMW DNA degradation and size analysis were performed and size profile using Y chromosome analysis

To study the degradation of gHMW DNA, we examined nuclear chromatin from PBMC during incubation in blood fluids. We observed a progressive decrease of total DNA concentration and long DNA fragments (> 246 bp) over the course of 24 h incubation in serum (Fig. 2A), whereas no change in terms of concentration and DII occurred to DNA from full blood or plasma collected in EDTA tubes (Fig. 2A). This showed that shorter fragments shorter than 246 bp did not exist or existed only in minimal quantities before incubation in serum, in contrast to during incubation in serum. More details of the results of this experiment are provided in Additional file 1: Table S2.

While no peak appears at the start of incubation, sWGS revealed as soon as following 5 min incubation the appearance of the peaks at 167 and 350 bp (Fig. 2B) which correspond to mono-N DNA and di-N DNA as found in cirDNA size profile of healthy individuals (Fig. 2C). The DNA size profile after 24 h incubation in serum showed an accumulation of short fragments below 160 bp (down to 41 bp), with the appearance of subpeaks with a ~ 10 bp periodicity revealing at that size range mononucleosome footprints (Fig. 2B). Thus, mono-N DNA accumulated up to 81.3% (Additional file 1: Table S2) during the 8 h incubation period in the 20–1000 bp size range. Whereas mono-, di-, and tri-N pattern appeared following 5 min incubation, di-N and tri-N DNA fractions decreased from 2 to 24 h incubation (Fig. 2B, Additional file 1:Table S2).

The use of serum shows here several limitations, given that the preparation of serum may lead to some cell degradation and possibly to the extracellular release of DNase or DNA, and the apparition of components linked to coagulation. While extracellularly released intracellular DNase should be diluted in a large amount of serum nuclease, and extracellular DNA release cannot interfere due to the specific quantification of Chr Y sequences, a bias may emerge as we cannot exclude the possibility of coagulation-derived components interfering with the DNA degradation rate, by interfering with DNA stability, for instance. Thus, the serum in which DNA degradation is examined is artificial, limiting the direct comparison of these observations to what occurs to the plasma in blood circulation.

### Kinetics of ex vivo NET production

**Fig. 3:**
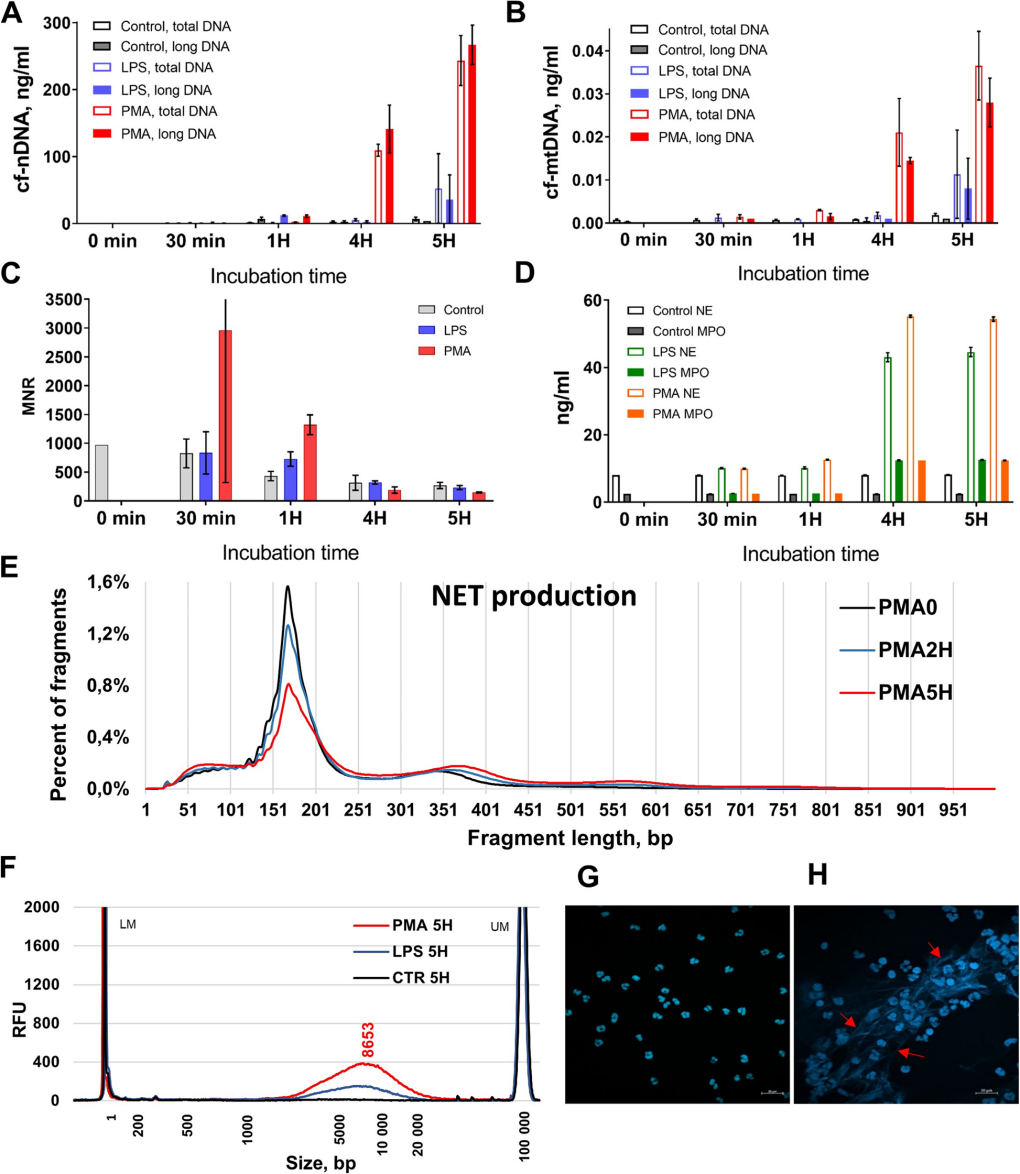
Kinetics of NET production in vitro. The kinetics of cfDNA, as determined by qPCR, in the supernatant of control non-stimulated and stimulated by 1 mg/ml LPS or 100 nM PMA human neutrophils from a healthy donor following incubation for up to 5 h at 37 °C in cell culture: **A** total and long (> 320 bp) nuclear cf-nDNA concentration; **B** total and long (> 310 bp) mitochondrial cf-mtDNA concentration; **C** MNR (ratio of mitochondrial to nuclear DNA concentration). **D** Kinetics of NE and MPO in the supernatant of the same control non-stimulated and stimulated by 1 mg/ml LPS or 100 nM PMA. Data are represented as mean ± SD. **E** NET production: the cfDNA size profiles of the supernatant of human neutrophils before incubation and stimulation (PMA 0), and following stimulation by PMA 100 nM and incubation at 37 °C in cell culture for 2 h (PMA 2 h) and 5 h (PMA 5 h), as determined by sWGS. **F** Analysis of cfDNA size profile by capillary electrophoresis (Fragment Analyzer) in the supernatant of control non-stimulated (CTR 5 h) and stimulated by LPS (LPS 5 h) and PMA (PMA 5 h) neutrophils following 5 h incubation in cell culture. **G, H** The fluorescent microscopic image of Hoechst stained neutrophils stimulated by LPS (**H**) and control neutrophils (**G**) following 5 h incubation in cell culture. Examination of DNA content using Y chromosome analysis

Ex vivo isolated neutrophils were stimulated by PMA or LPS, in order to investigate NET-associated DNA release (Fig. 3). The qPCR analysis revealed a sharp increase of cf-nDNA and cf-mtDNA concentrations in the supernatant of stimulated neutrophils, with minor increase in control cells (Fig. 3A,B). After 5 h incubation, the total cfDNA concentrations were 7–35 fold higher in the supernatant of LPS- and PMA-stimulated neutrophils than in the supernatant of non-stimulated controls. NE and MPO concentration increased up to fivefold in the supernatant of stimulated neutrophils as compared to unstimulated control after 5 h incubation in cell culture (Fig. 3D). The Pearson correlation study revealed strong positive correlations between DNA and NET protein (NE, MPO) markers in stimulated neutrophils (Additional file 1: Fig. S3). The kinetics of the release in the cell culture supernatant of MPO, NE, cf-nDNA, and cf-mtDNA are all remarkably similar up to 5 h incubation, suggesting the co-occurring release of these components, and consequently suggesting that cfDNA may be a byproduct of NET.

Fragment size profile analysis by sWGS of DNA from the supernatant of ex vivo stimulated neutrophils (Fig. 3E) revealed the typical chromatin organization pattern as observed for cirDNA derived from human blood (Additional file 1: Fig. S4). Overall, there was a progressive increase of di-N, tri-N, and long DNA-associated peaks (Fig. 3E; PMA 2 h, PMA 5 h), as compared to baseline (PMA 0) (Fig. 3E). Notably, while the mono-N population peaked at 167 bp irrespective of incubation time, the di-N DNA peaked at 365 and at 341 bp in PMA 5 h and baseline (PMA 0), respectively. In addition, PMA 5 h stimulated neutrophils supernatant showed a small third peak at 560 bp, corresponding to tri-N DNA. Capillary electrophoretic analysis demonstrated that activated neutrophils produced HMW DNA whose mean size was 8600 bp. In contrast, non-stimulated neutrophils did not show an accumulation of long DNA fragments even after 5 h of incubation in cell culture (Fig. 3F).

To visualize the NET structure, we performed a DNA specific Hoechst staining of stimulated (Fig. 3H) and control neutrophils (Fig. 3G) with presence of NET fibers observed around the stimulated neutrophils.

### Kinetics of in vitro NET degradation in blood

**Fig. 4:**
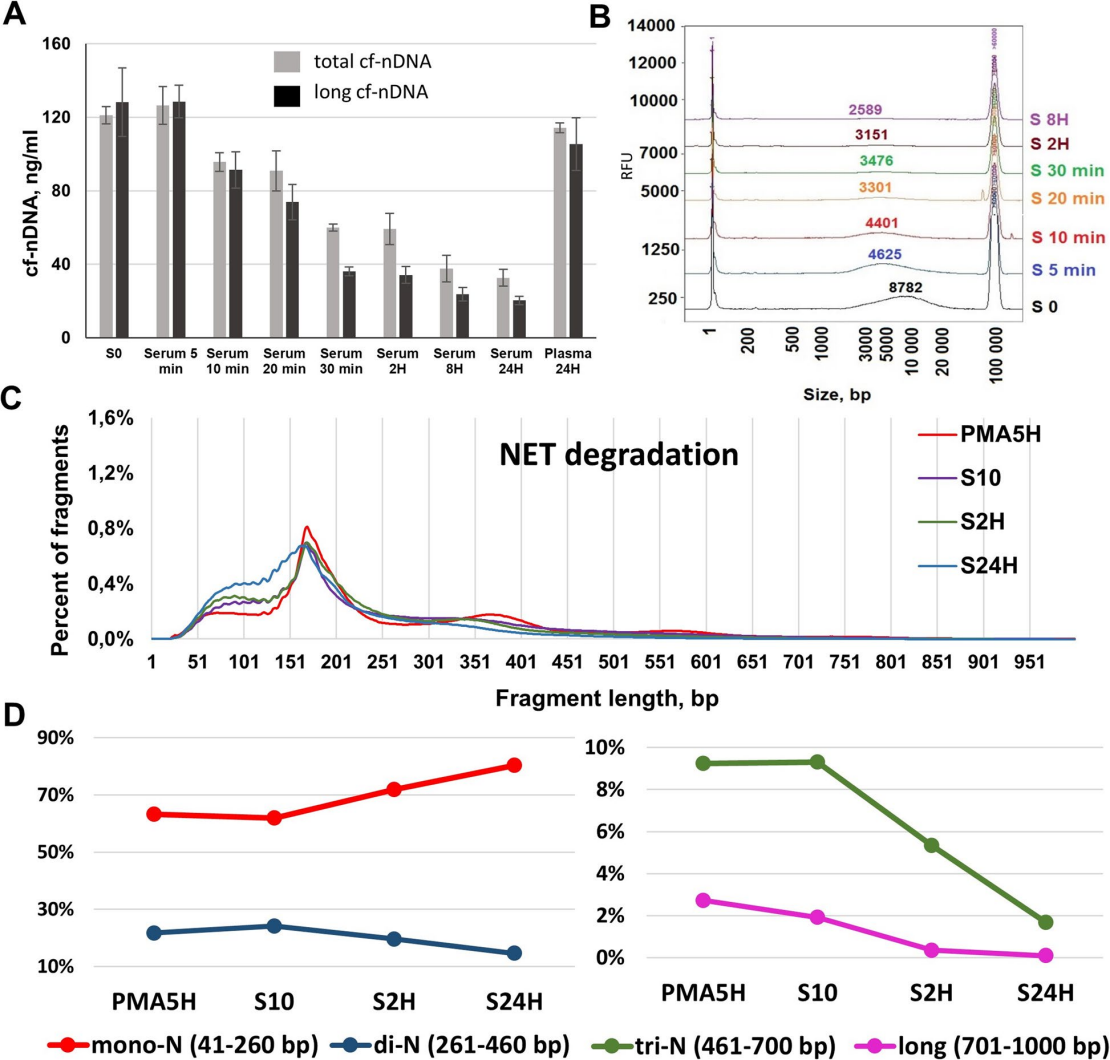
Kinetics of NET degradation in blood fluids. Kinetics of total and long (> 246 bp) nuclear cell-free DNA (cf-nDNA) concentration following the degradation of NET (supernatant of PMA-activated neutrophils) by incubation in serum/plasma up to 24 h: before incubation (S0) and after incubation at 37 °C in plasma for 24 h or in serum for 5 min/10 min/20 min/30 min/2 h/8 h/24 h. Data are represented as mean ± SD. **B** Analysis of cfDNA size profile by capillary electrophoresis (Fragment Analyzer) in the supernatant of PMA-stimulated (5 h) neutrophils (S0) and following its incubation in serum (S 5 min/S 10 min/S 20 min/S 30 min/S2H/S8H). **C** NET degradation in serum: the cfDNA size profiles of the supernatant of PMA-stimulated (5 h) neutrophils (PMA 5 h), and following incubation of this supernatant in serum at 37 °C for 10 min (S10), 2 h (S2H), and 24 h (S24H). **D** Analysis of sWGS data for assessment of the cfDNA fractions corresponding to mono-, di-, and trinucleosome-associated fragments during NET degradation. Examination of DNA degradation and of size profile were performed using Y chromosome

To study NET degradation, we placed the culture medium of 5 h PMA-stimulated neutrophil obtained from a male donor in the plasma and serum of a healthy female donor. The concentrations of both total cfDNA and long cfDNA fragments decreased progressively in serum, with the cf-nDNA concentration after 24 h incubation in serum being 3–4 fold lower than the cfDNA concentration at baseline (S0) (Fig. 4A). NET were seen to undergo rapid and strong degradation, with a significant loss of 28, 48, and 71% of NET-derived DNA input being observed after 10 min, 30 min, and 24 h. The DII decreased from 1.0 to 0.6 during the first 30 min, then stabilized up to 24 h (Additional file 1: Fig. S2). Incubation of NET in plasma for 24 h did not lead to a significant change of the concentrations of total DNA or long DNA fragments (Fig. 4A), or of the DII (Additional file 1: Fig. S2). Capillary electrophoresis demonstrated the shortening and degradation of long DNA fragments during 8 h incubation in serum: the average size of long DNA fragments decreased from 8782 to 2589 bp, with the fraction of DNA fragments in the 1–10 k bp range decreasing from 86 to 36% (Fig. 4B). Note, only < 2% and 1% of this fragment length population remained following 2 and 24 h incubation.

sWGS fragment size profile analysis of NET-derived DNA, performed in the course of incubation in serum, revealed an evolution of the organization pattern of cfDNA-associated chromatin. We observed a progressive accumulation of DNA fragments < 167 bp, and a decrease of the di-N associated peak (260–460 bp) from 10 min to 24 h incubation in serum (Fig. 4C). In contrast to the effect on NET production in culture medium (Fig. 3E), NET degradation in serum produced a shortening of the di-N DNA, with the maximum associated peak decreasing from 365 to 321 bp (Fig. 4C). Furthermore, the tri-N DNA fragment population disappeared following 24 h incubation in serum (Fig. 4C).

When comparing the size profile analysis of DNA extracted during the incubation of stimulated neutrophils (NET production) and of DNA extracted in the course of incubation in blood fluids (NET degradation), we observed that (i) NET production was associated with the increase of fractions of mono-N, di-N, tri-N, and long DNA fragments up to 65, 22, 9, and 2.7%, respectively. In contrast, NET degradation in serum led to an accumulation of the mono-N DNA fraction representing 80%, and a decrease of the di-N, tri-N, and long DNA fractions down to 15, 1.7, and 2.73 to 0.10%, respectively (Fig. 4D). The high, intermediate, and low degradation rates of long, tri-N, and di-N DNA are in contrast to the increase of mono-N DNA, suggesting that DNA in oligonucleosomes are degraded to mono-N DNA. The mono-N DNA amount corresponds to a balance between the respective rates of their degradation and their accumulation due to DNA lower nuclease sensitization when packed in mononucleosomes. When combining capillary electrophoresis and sWGS data, it appears that high molecular weight DNA from NET chromatin fibers are highly degraded, resulting in the indirect detection of oligonucleosomes, principally mononucleosomes.

### gHMW DNA degradation in presence of NE and MPO

**Fig. 5:**
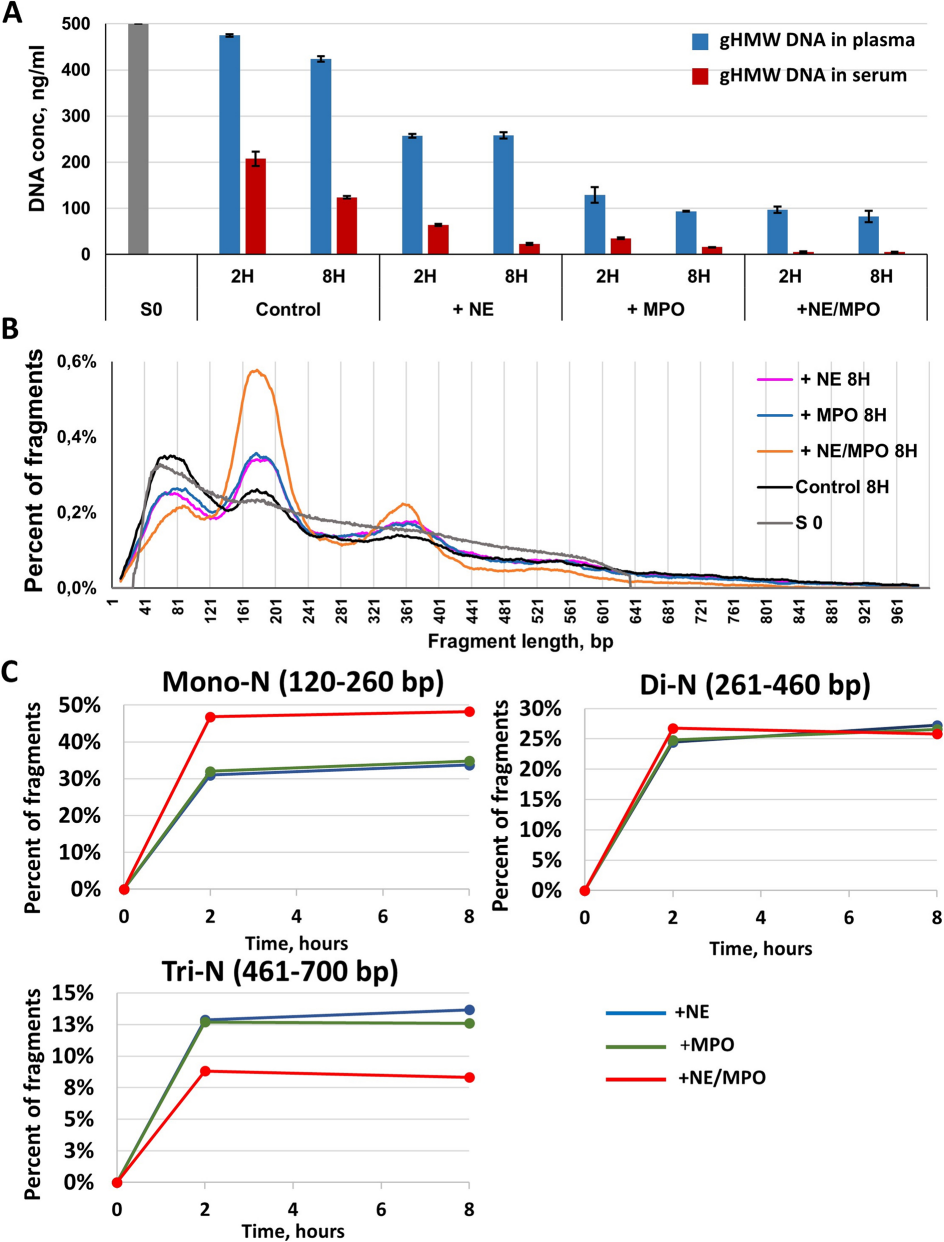
gHMW DNA degradation in presence of NE and MPO.** A** Quantification of total DNA by qPCR in cell lysate containing gHMW DNA before degradation (S0) and following gHMW DNA incubation at 37 °C for 2 or 8 h: in plasma or serum (control) and in plasma or serum in the presence of neutrophil elastase (+ NE), myeloperoxidase (+ MPO) or both (+ NE/MPO). **B** CfDNA size profiles for gHMW DNA samples before incubation in blood fluids (S0) and following 8 h of incubation at 37 °C in serum: control 8 h or in the presence of neutrophil elastase (+ NE), myeloperoxidase (+ MPO), or both (+ NE/MPO). **C** Analysis of cfDNA fractions corresponding to mono-, di-, and trinucleosome (Mono-N, Di-N, and Tri-N, respectively) associated fragments as determined by sWGS: cfDNA fractions in the course of gHMW DNA degradation in the presence of neutrophil elastase (+ NE), myeloperoxidase (+ MPO), or both (+ NE/MPO). Examination of gHMW DNA degradation and of size profile were performed using Y chromosome analysis

As quantified by qPCR, after 2 h incubation in serum containing NE or MPO, the amount of gHMW DNA showed a 3- and fivefold decrease, respectively, as compared with control incubation (64 ng/ml and 35 ng/ml vs 207 ng/ml, respectively) (Fig. 5A).

We observed an even more significant difference after 8 h incubation, as the presence of NE and MPO led to 5.4-fold and eightfold decrease, respectively, of total DNA concentration, as compared to control incubation (23 and 16 ng/ml vs 124 ng/ml) (Fig. 5A). These variations appeared even more impressive with the simultaneous addition of both NE and MPO, with 8 h incubation leading to a 25-fold decrease of total DNA concentration, as compared to control incubation (5 ng/ml vs 124 ng/ml) (Fig. 5A). ~ 99% of gHMW DNA were degraded following the later condition.

As shown in Fig. 5A and in Additional file 2, the estimated degradation rate within the first 2 h and from 2 to 8 h incubation is 11.6- and 4.9-fold higher in serum than in plasma, respectively. It appears that within the first 2 h of incubation the degradation rate is 3- and fourfold higher than that observed during the 2–8 h incubation period for controls and NE/MPO containing serum. The estimated degradation rate in serum or in plasma is always significantly higher in NE/MPO containing serum than in controls, irrespective of whether the first 2 or 2–8 h incubation periods are considered. For instance, data showed 1.5-, 1.6-, and 1.8-fold increase in NE, MPO, and NE + MPO in serum, respectively; similarly, albeit to a larger extent, ~ 10-, 15-, and 16-fold increases were shown in NE, MPO, and NE + MPO in plasma, respectively. Whereas the degradation rate is 11.6-fold higher in serum as compared to plasma in controls during the first 2 h, it is much more similar when adding NE, MPO, and NE + MPO (1.8-, 1.3-, and 1.2-fold, respectively). The highest estimated DNA degradation rate (4.12 ng/mL/minutes) was found in NE + MPO containing serum during the first 2 h of incubation. Note, there is only a slight increase in the degradation rate in serum vs plasma during both incubation time periods when MPO and NE + MPO are added (~ 1.2-fold in both 0–2 h and 2–8 h time periods, respectively) and when NE is supplemented to serum and plasma (~ 1.8- and twofold, in 0–2 and 2–8 h time periods, respectively). This is a striking demonstration of the nearly equivalent DNA degradation rate deriving from NE, and to a greater extent deriving from MPO activities, irrespective of nuclease inhibition. gHMW DNA degradation rate for each condition of incubation is described in Additional file 2.

From the data shown in Fig. 5A, we can estimate the capacity of NE and/or MPO to affect the gHMW DNA degradation rate. Data suggest that (i) the addition of NE and/or MPO to plasma or to serum greatly increases HMW DNA degradation (Fig. 5A); (ii) NE and MPO independently increase HMW DNA degradation; (iii) NE and/or MPO greatly increase DNA degradation in the presence of high nuclease activity as well as when nuclease activity is strongly inhibited; (iv) MPO shows an increase in DNA degradation approximately twice that of NE; and (v) NE and MPO may act separately to contribute to DNA degradation, while there is only a slight increase when NE and MPO are combined. The gHMW DNA degradation rate for each condition of incubation is described in Additional file 2.

We also observed a decrease of gHMW DNA during incubation in plasma. But these variation levels were not as pronounced as in the case of gHMW DNA incubation in serum (Fig. 5A).

The fragment frequency as determined from the size profile of gHMW DNA before incubation in serum decreased linearly from 50 to 700 bp (Fig. 5B), which was similar to what had been observed previously (Fig. 2B). After incubation in serum, the gHMW DNA fragment size profile showed the specific chromatin organization pattern with peaks corresponding to mono-N, di-N and (less prominently) tri-N peaks. All three peaks were more pronounced when gHMW DNA was degraded in the presence of NE or MPO than in serum only (control sample). Degradation with the combined incubation of both NE and MPO was associated with the most prominent peaks of mono-N and di-N (Fig. 5B). gHMW DNA incubation in serum for 8 h (Control 8 h) led to a clear modification of the size profile, as determined by sWGS. Incubation in serum with NE or MPO alone for either 2 or 8 h provoked an equivalent increase of mono-N and di-N DNA populations. Incubation in serum with NE and MPO combined further enhanced the prominence of both corresponding peaks (Fig. 5B). As shown in Fig. 5C, the increase of the mono-N DNA fraction is faster within the first 2 h of incubation than that observed during the 2–8 h incubation period (Fig. 5C), mirroring the higher cirDNA degradation rate estimated during the first 2 h of incubation.

### In vivo study on the role of NE in cirDNA fragmentome

In order to elucidate the role of NE in DNA degradation, we performed an in vivo study of cirDNA in WT, NE KO, and alpha antitrypsin (AAT) KO mice [40]. EDTA blood samples were collected from these 3 groups of mice (10 mice in each group), and plasma was isolated for further NE and cirDNA analysis. Quantification of NE by ELISA confirmed the absence of NE in NE KO mice and showed that the concentration of NE was significantly higher in AAT KO mice than in WT mice (2.52 ± 0.39 ng/ml in WT vs 3.31 ± 0.67 in AAT KO, *p* = 0.005).

**Fig. 6:**
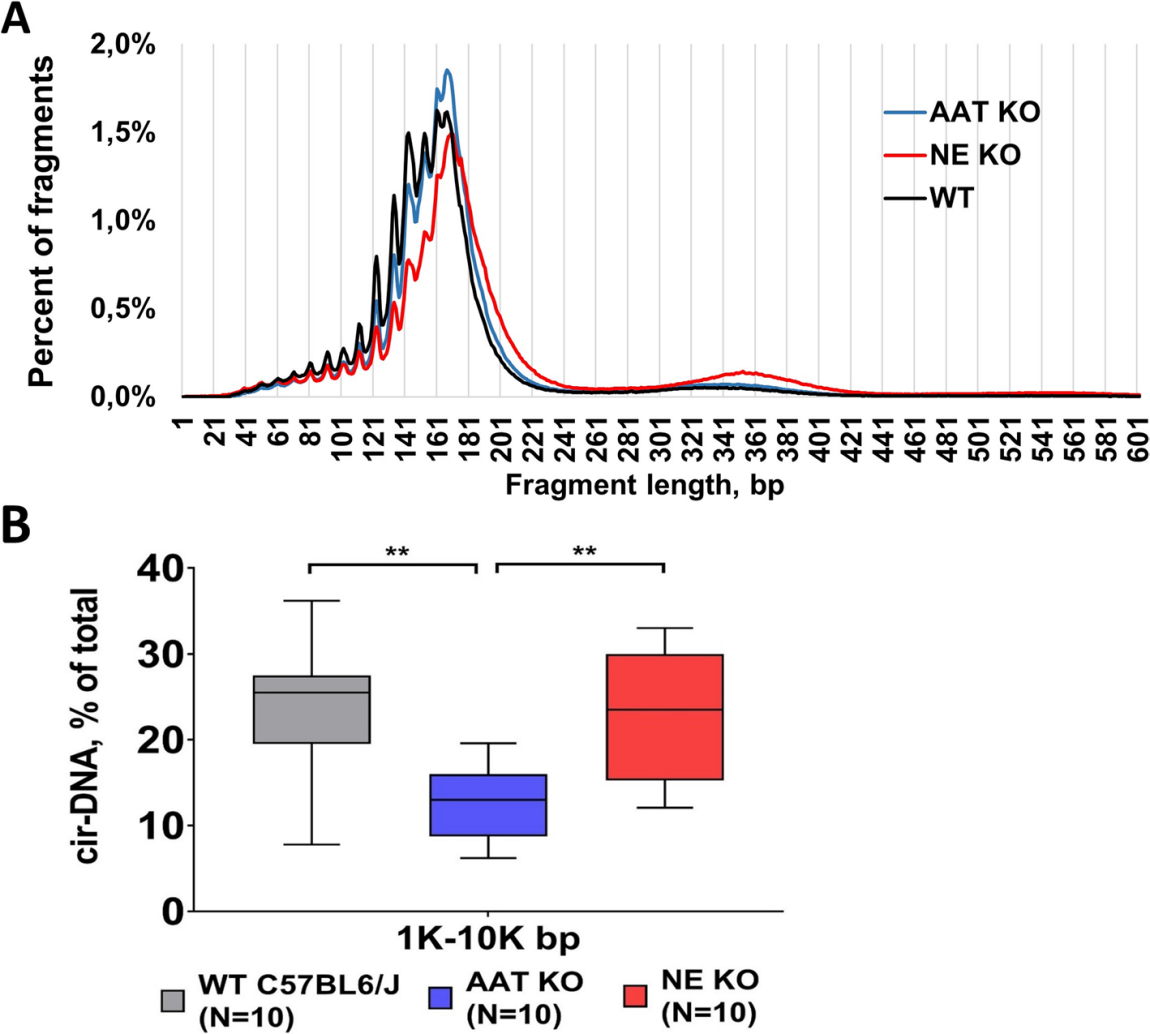
In vivo study on the role of the NE in cirDNA fragmentome.** A** Illustrative size profiles of cirDNA from plasma of wild type mice (WT), neutrophil elastase knockout mice (NE KO), and mice knockout for alpha-1 antitrypsin (AAT KO), as determined by sWGS. **B** Analysis of long fragment cirDNA fraction in plasma of WT, NE KO, and AAT KO, as determined by capillary electrophoresis (Fragment Analyser, Agilent)

The cirDNA size profiles in plasma of WT, NE KO, and AAT KO mice were determined by sWGS (Fig. 6A). As observed in human blood, the cirDNA fragment size profile of each group of mice (*N* = 10) revealed the mammalian chromatin organization, with the main mono-N population peaking at 167 bp, and the second, less prominent population peaking at 335–355 bp, which corresponds to di-N. The WT and AAT KO mice plasma showed similar profiles, with a major population peaking at 167 bp and at subpeaks with ~ 10 bp periodicity below 180 bp, and with a minor population peaking at 335 bp. The cirDNA size profile of NE KO mice differed from that of the WT and AAT mice, exhibiting a shift to a longer average length of cirDNA fragments. Detailed descriptions of the size profile characteristics of the three types of mice are presented in Additional file 1:Fig. S5.

Analysis of cirDNA fractions by capillary electrophoresis revealed a significantly decreased fraction of long DNA fragments in AAT KO mice: the fraction of 1–10 k bp fragments was twofold higher in WT and NE KO mice than in AAT KO mice (Fig. 6B).

### NET markers and cirDNA association in plasma in several disorders

**Fig. 7:**
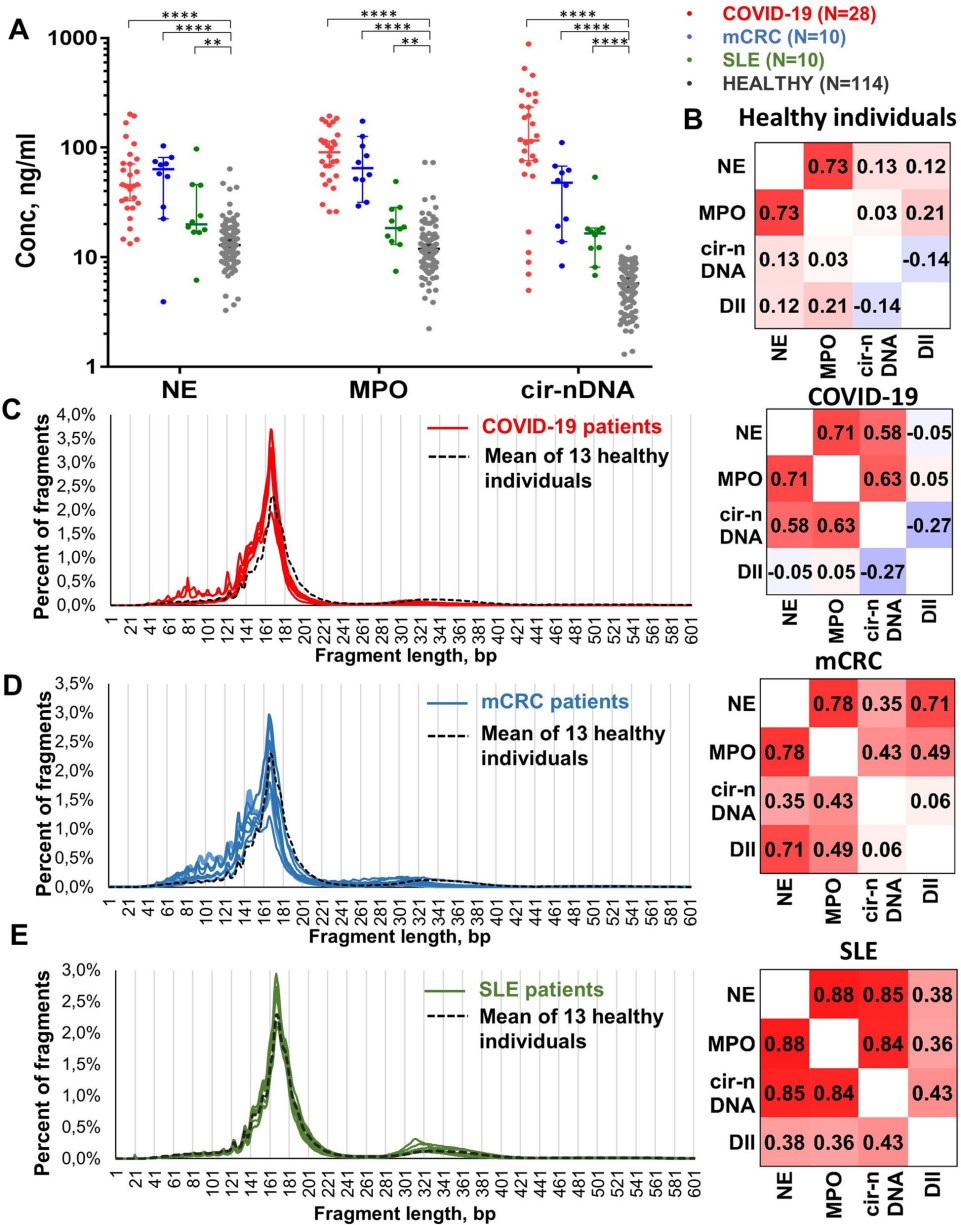
NET association with several disorders. **A** Comparison of cirDNA, MPO, and NE concentrations (ng/mL of plasma) in COVID-19 patients (*n* = 28), SLE patients (*n* = 10), mCRC patients (*n* = 10) and healthy individuals (HI) (*n* = 114). Lines represent median with 95% CI. Mann–Whitney test was performed to compare values for cirDNA, MPO, and NE in mCRC patients and in HI. A probability of less than 0.05 was considered statistically significant; **p < 0.01, ****p < 0.0001. Each dot represents the values of a single patient or a single healthy individual. CirDNA: circulating cell-free DNA; MPO: myeloperoxidase; NE: neutrophil elastase. **B** Correlation matrix of MPO and NE concentrations (ng/mL plasma) with cirDNA parameters in healthy individuals (*n* = 114). Heatmap manifests the strength of the relationship by Pearson’s correlation analysis. Cir-nDNA: circulating cell-free DNA of nuclear origin; MPO: myeloperoxidase; NE: neutrophil elastase; DII: DNA integrity index; cir-mtDNA: circulating cell-free DNA of mitochondrial origin; MNR: ratio of mitochondrial to nuclear circulating DNA concentration. Correlation matrix of MPO and NE concentrations (ng/mL plasma) with cirDNA parameters and comparison of the cfDNA size profile determined by sWGS of the healthy individual mean (black dashed line) and **C** COVID-19 patients, **D** metastatic colorectal cancer (mCRC) patients, and **E** systemic lupus erythematosus (SLE) patients

Plasma NE, MPO, and cirDNA concentrations were significantly elevated in the SLE, mCRC, and COVID-19 patients compared to HI (Fig. 7A). The highest values of NET markers were found in the plasma of severe COVID-19 patients, which showed significant differences from HI in the analysis of NE (45.3 ng/ml vs 12.88 ng/ml, *p* < 0.000001), MPO (90.85 ng/ml vs 11.91 ng/ml, *p* < 0.000001), and cirDNA (116 ng/ml vs 5.76 ng/ml, *p* < 0.000001). There was no correlation between cir-nDNA and NE/MPO concentration in HI (Fig. 6B), while they positively correlated in SLE, mCRC, and COVID-19 patients (Fig. 7C–E). The highest positive correlations of cirDNA with NE and MPO were found in the plasma of SLE patients (*r* = 0.85 and *r* = 0.84, respectively) (Fig. 7E).

As determined by sWGS, the cirDNA size profiles of SLE, mCRC, and COVID-19 patients was somewhat similar to those of HI, which clearly points to mononucleosomes as the predominant structures from which DNA obtains a degree of stability. However, the size profiles of cirDNA from COVID-19 and mCRC patients show a clear shift to shorter fragments, as compared to HI. The di-N associated DNA fragment population from healthy plasma peaked at 332 bp, while it did so at ~ 310–320 bp for SLE, mCRC, and COVID-19 patient plasma. The size profile of SLE patients was relatively homogenous and similar to that of HI, except for its di-N peak.

## Discussion

Sufficient evidence now exists regarding NET as one (previously unappreciated) source of cirDNA to indicate their strong potential as a source of biomarkers for various inflammatory disorders and other pathologies. However, no study to date has directly proven that either chromatin or NET DNA fibers degrade into mono-N DNA which could be released into the blood circulation without implying cell death.

### The gHMW DNA can be degraded in serum into mononucleosomes

For more than two decades, the prominence of mononucleosomes in cirDNA extract encouraged the postulation that apoptosis was the main mechanism of release into blood circulation [4, 7, 41]. Previous efforts to study genomic DNA degradation, however, offered conflicting conclusions regarding the role of apoptosis and apoptotic nucleases in internucleosomal DNA fragmentation and the production of mononucleosomes. On the one hand, certain studies report that cirDNA originates in the apoptotic DNA fragments generated by caspase-activated DNase (CAD), because the cfDNA fragment sizes generally approximate to a mononucleosomal size [42]. Koyama et al. [14] propose that cirDNA can originate from both apoptotic and necrotic cells and that CAD, DNase γ, and DNase I are responsible for the generation of DNA fragments. On the other hand, a different study showed that apoptosis in CAD-deficient mice caused DNA fragmentation equivalent to that in WT mice; thus concluding that apoptosis-associated CAD is therefore not essential for DNA degradation in vivo, they suggested that phagocytes are rather as the main actor in DNA fragmentation [43]. Finally, yet another study in mice has shown that, in apoptosis, CAD is insufficient for degrading chromatin and requires DNase1L3 to degrade it into mononucleosomes [44]. Given these contradictory data and the uncertain role of apoptosis in DNA fragmentation in cell culture or in vivo in mice, we preferred to focus on the general ability of human blood to fragment DNA regardless of the mechanisms of cell death [7]. Our current study first investigated DNA degradation by adding gHMW DNA from a cell lysate to the serum or plasma of a HI under physiological conditions meaning that no cells (and thus no apoptosis or any other cell death events) could be involved in DNA degradation. Our study design implies the use of gHMW DNA and blood donors of opposite genders, and explores the gHMW DNA topology under degradation by examining the *SRY* sequence. An accumulation of DNA fragments associated with mononucleosomes showed that, in the absence of apoptosis, exposure to serum nucleases alone is sufficient for the effective degradation of gHMW DNA down to mono-N DNA fragments. Data also confirmed mono-N as the most stabilized structure in blood with which cirDNA can associate [31, 45].

In previous studies, we and other groups have scrutinized the size distribution of cirDNA in the plasma of HI and cancer patients [31, 45]. Among those findings, a sWGS assay revealed the predominance of chromatosome/mononucleosome-associated cirDNA fragments and the presence in the plasma of HI and cancer patients of a minor population corresponding to dinucleosome-associated cirDNA. A combination of double- and single-strand DNA library preparations (DSP and SSP) and nested qPCR (N-qPCR) enabled the estimation of the wide size distribution of cirDNA fragments inserted in mononucleosomes, di-nucleosomes, and chromatin of higher molecular size (> 1000 bp) as 67.5–80%, 9.4–11.5%, and 8.5–21.0%, respectively [31]. We found a striking reproducibility of the cirDNA size profiles in HI [31], in that the maximal size and frequency of the major peak and all subpeaks varied by merely 1 or 2 bp and by 2–20%, respectively.

In the present study, we observed a characteristic chromatin pattern after gHMW DNA degradation by serum nucleases, in which the most prominent peak is of mono-N cfDNA, and the less prominent peak of di-N cfDNA (Fig. 2B) as observed for cirDNA in vivo. We observed a very rapid degradation and the depletion of long DNA fragments resulted in the formation of oligonucleosomes, making them the main substrate for DNase attacks, and leading to the subsequent accumulation of mono-N and di-N (after 2 h incubation). This also led to the accumulation of short DNA fragments in the 40–160 bp range, indicating the dynamic action of serum nucleases on the internucleosomal DNA, the DNA linked to the histone H1, and the 14 DNA base pairs exposed at the surface of the nucleosome core particle at a ~ 10 bp periodicity [31, 45, 46].

As determined by sWGS, the analysis of the DNA fragment fractions corresponding to mono-N, di-N, and tri-N confirms these conclusions and adds to our quantitative assessment of the kinetics of gHMW DNA degradation in serum. When comparing the proportions of mono-N, di-N, or tri-N DNA, data revealed the higher resistance of mononucleosomes to DNase attacks, as compared to oligonucleosomes. We therefore conclude that in normal physiological conditions blood might be capable of degrading gHMW DNA, and especially nicking and degrading the internucleosomal DNA; this leads to the accumulation of mononucleosomes/chromatasomes.

### Activated neutrophils produce HMW DNA fragments ranging from 1500 to 30,000 bp

Although there are several reports based on the visualization of NET in vitro by fluorescent microscopy, which demonstrates that NET consist of the DNA fibers, no studies have analyzed the structure and length of DNA released by activated neutrophils and its association with cirDNA production.

In our present study, we describe the accumulation of HMW DNA fragments and the increase of NET markers in the supernatant of ex vivo activated neutrophils. This would indicate the formation of these DNA fibers decorated with NET-specific proteins, such as NE and MPO. Our data confirms these results by revealing the significant correlations of the DNA markers (based on size and amount) with MPO and NE. These results show the association of extracellular DNA with NET and suggest that NET are the source of the dynamic degradation undergone by HMW DNA in blood.

The MNR negatively correlated with the concentration of NE and MPO and decreased with the activation of the neutrophils (Fig. 3C and Additional file 1: Fig. S3). In previous studies, we have already described the decrease of MNR [26, 35, 47] and the increase of NE and MPO in cancer plasma, as compared to healthy individuals [26]. We believe this to be linked to the activation of neutrophils and the release of NET and could be explained by the structural differences between nuclear and mitochondrial DNA. The release of mitochondrial DNA (mtDNA) by activated neutrophils has been detected in several studies [48, 49]. Being devoid of an association with histones (and the protection which results from that), mtDNA is more subject to fragmentation than nuclear DNA, when not inside the circulating mitochondria [50]. We can therefore suggest that the rapid and massive production of both nuclear and mtDNA, combined with the faster degradation of mtDNA compared to the relatively stable nDNA (protected by the mononucleosome structure), leads to an imbalance in the proportions of cir-nDNA and cir-mtDNA, and thus to a decrease of MNR.

### The in vitro degradation in serum of the long DNA fragments originating from ex vivo generated NET leads to the accumulation of the mononucleosome-associated fraction of cfDNA

qPCR quantification revealed the progressive decrease of the total DNA and the long DNA fragment concentrations (Fig. 4A), as well as the synchronous decrease of the DII (Additional file 1: Fig. S2), confirming our postulate that DNA from NET could be degraded by serum nucleases down to mono-N fragments. Analysis of the same DNA extracts by capillary electrophoresis demonstrated the shortening and dynamic reduction of the 1–30 kb DNA fraction during incubation in serum (Fig. 4B). That observation agrees with the qPCR results. The analysis of DNA by capillary electrophoresis does not allow us to distinguish male and female DNA using the sequence of the Y chromosome and therefore to directly quantify their respective amount. However, given the 30-fold excess of DNA from NET (0.36 ng/μL) compared to cirDNA from serum (0.012 ng/μL), we can assume that more than 95% of the DNA fragments detected by capillary electrophoresis originate from NET.

The NET production was associated with the accumulation of di-N and tri-N DNA. Conversely, in the course of the degradation of NET DNA in serum we observed an inversion of all those proportions, with the progressive disappearance of di-N and tri-N associated DNA fragments, and the accumulation of mono-N DNA fragments (Fig. 4D). These results confirm our hypothesis that, when expelled to the extracellular milieu (in this case, the serum), the long fragments of DNA originating from NET are predominantly degraded to mononucleosomes, given that (of all NET byproducts) these constitute the most stabilized structure with which cirDNA can associate.

The rate of DNA degradation of NET and gHMW DNA can in no way be compared to previous observations about the half-life of cirDNA in the circulation, where pharmacological aspects such as clearance, mononucleosome phagocytosis, or metabolic pathways are heavily involved. Our main objective is to show that the degradation of NET or chromatin leads to mono-N cirDNA. In physiological conditions, detectable cirDNA results from the balance between extracellular DNA release and elimination, as above mentioned. Extracellular release may result from various mechanisms. Of these, NET could be a significant source, along with other mechanisms such as apoptosis, necrosis, active cellular release, or extracellular traps generated from other blood cell types [25].

Chromatin organization along the genome is not random and may be specific to cell gene regulation and cell types [51, 52]. CirDNA cell-of-origin was recently investigated using WGS and fragmentomics. Analytical signals from the cirDNA fragmentome include non-random fragmentation pattern, transcription factor occupancy, GC contents, and end motif pattern, and by extension methylation. Studies from Y. Dor’s team based on cirDNA methylome analysis [53, 54] appear to indicate that the main cirDNA cells-of-origin are megakaryocytic and lymphocytic (especially neutrophilic). More recent reports confirmed that among a great variety of cells-of-origin, the neutrophil origin appears to be one of the most important [55]. Interestingly, this report and others also found that the cell-of-origin proportion may vary when comparing plasma of HI and non-HI, particularly cancer patients. In addition, cirDNA fragmentation analysis in open chromatin regions [34] and nucleosome occupancy also informs about tissue of origin. Thus, Shendure’s milestone study [46] revealed that lymphoid or myeloid origins have the largest proportions consistent with hematopoietic cells as the dominant source of cirDNA in HI, in contrast to cancer patients. This opens up a wide range of opportunities for diagnosis and the interrogation of physiological and pathological processes [53, 56].

### NE affects the cirDNA-associated structure in vivo by enhancing DNA fragmentation

NE was found to be involved in DNA decondensation in the nuclei of activated neutrophils [27]. It could therefore be suggested that NE contributes to DNA fragmentation. In our in vivo study of cirDNA deriving from the plasma of WT, NE KO, and AAT KO mice, we demonstrated that the cirDNA size profiles revealed by sWGS differ according to NE activity. The absence of NE (in NE KO mice) was associated with the increase of the di-N associated cirDNA fragments and a significant increase of the average fragment size, which would indicate impaired DNA fragmentation, as compared to WT mice. The cirDNA fragment size profile of WT and AAT KO mice were visually similar, despite the increased activity of NE in the AAT KO mice. Capillary electrophoresis analysis revealed a decrease in the 1–10 k bp cirDNA fragment fraction in AAT KO mice, suggesting that NE facilitates or participates in the degradation of DNA fragments of sizes equivalent to those generated from ex vivo PMA-stimulated neutrophils. Taken together, these results permit the conclusion that NE affects the cirDNA structure and, consequently, the fragmentation features of cirDNA which originates from NET.

### NE and MPO synergistically catabolize NET

The mechanism of cirDNA generation, in particular its enzymatic activity, remains insufficiently understood. Theoretically, the catabolism of DNA which leads to cirDNA production may be enabled by nucleases either intracellularly (DNase1L1, DNA fragmentation factor B or DFFB, DFFB/CAD and endonuclease G) or extracellularly (DNase1 and DNase1L3 or DNase γ). The paradigm in which, historically, this mechanism has been understood involved mainly apoptosis as well as necrosis [4, 7]. This is due to initial observations of laddered patterns of oligonucleosomal-size pieces (being the hallmarks of canonical apoptosis) by gel electrophoresis of cirDNA extract, and due also to the more recent characterization of the mononucleosome structure, principally by sequencing and fragmentomics [31, 45, 46, 57]. The main nuclease responsible for the internucleosomal double-strand (ds) cleavage of genomic DNA is DFFB/CAD; it may also be responsible, however, for single-strand breaks (ssB) during apoptosis [58]. Watanabe et al. observed that DNase1L3 cooperated with caspase-activated DNase (CAD) in the case of anti-Fas-mediated hepatocyte apoptosis, while DNase1L3 was responsible only for the generation of cirDNA in acetaminophen-induced hepatocyte necrosis. However, they were unable to detect cirDNA in CAD − / − DNase1L3 − / − mice, indicating that DNase1 is not sufficient for the generation of cirDNA following apoptosis [44]. This supported the observations made by D. Lo’s group [59, 60], who reported similar cirDNA levels in both DNase1 − / − and WT mice in steady-state condition. Although the mechanism of DNase activity is not fully understood, it shows correlation with inflammation; for instance, its induction mechanism may be associated with macrophage activation [61]. Collating various observations [14, 44, 60, 62] provides clues that CAD plays a key role in the initiation of cell death, by producing dsDNA breaks intracellularly, while DNase1L3 and DNase1 (both induced by apoptosis and necrosis) pursue chromatin catabolism extracellularly, such as in blood circulation. DNase1 is expressed by non-hematopoietic tissues and preferentially cleaves protein-free DNA [63, 64]. DNase1L3 (also known as DNase gamma) is secreted by immune cells and targets DNA–protein complexes such as nucleosomes [61, 64]. Note, several reports have postulated the role of active release in the production of DNA associated to extracellular vesicles [65]. The literature showed discrepancies about DNA being incorporated onto or into microvesicles. DNA encapsulated in vesicles would be less sensitive to nuclease attack, potentially modifying DNA fragmentation rate and process. Conversely, it was demonstrated that nuclease DNA1L3 could degrade DNA in microvesicles [61]. Regardless of these considerations, once released from vesicles its fragmentation process should be similar to that of cirDNA not associated with extracellular vesicles.

Over the last 5 years, we [3] and several other authors [44, 66, 67] have speculated about the role of NETosis in the generation of cirDNA. There are conflicting observations as to the nature of the nuclease-derived enzymatic activity involved in the degradation of NET to cirDNA. Both DNase1 and DNase1L3 have been shown to degrade NET in blood circulation [67, 68]. Jimenez et al. [67] showed that the toleration of chronic neutrophilia (as well as the prevention of vascular occlusion by NET clots during that process) requires both enzymes.

Data suggests that DNA degradation is increased when NE and/or MPO is added to serum nucleases and that DNA degradation is increased to a much larger extent in plasma when nuclease activity is greatly inhibited. Data strikingly showed that, in serum and in plasma, the DNA degradation rate due to NE was nearly equivalent, as it was (to a higher extent) due to MPO activities, irrespective of nuclease inhibition by EDTA as a chelating agent. Note, NE activity was found not dependent of Ca and Mg ions and determined in plasma [69]. Whereas MPO is dependent on Ca ions, its activity was still present in plasma [70]. This was the first direct observation of NE and MPO DNA degradation activity. Overall, our study consequently suggests that NE and MPO contribute to DNA catabolism. In addition, our data clearly demonstrated, for the first time, that NE and MPO contribute to and favor the DNA degradation process by releasing oligonucleosomes, mainly mono-N from NET. In addition, we suggest that, following neutrophil stimulation principally by oxidative explosion and its translocation to the nucleus, NE is continuously released from extracellular NET-associated granules, and continuously decondenses the chromatin. It has been established that histones are cleaved by NE derived from granulation and are citrullinated by peptidyl arginine deiminase 4 (PAD4) during intracellular NET formation. In the initial phase of this process, NE degrades the linker protein H1 and then the core histone proteins, in a process which is known to be enhanced by MPO [27]. MPO produces oxidizing agents such as hypochlorous acid (HOCl) from hydrogen peroxide (H_2_O_2_) and chloride anion (Cl −). Thus, we might postulate another possible hypothesis: oxidizers damage DNA, thus making it more susceptible to degradation by nucleases [71]. Drugs producing oxidizers might therefore be conceived as NET inhibitors, in pathologies where NET formation is uncontrolled [25].

In addition, our study first revealed that the synergistic association of NE and MPO contributes to the degradation of in vitro gHMW DNA, leading to the release of oligo- and principally mononucleosomes in serum containing medium. However, our study is limited since it cannot fully establish whether NE/MPO contribute to NET degradation either directly (such as having nuclease activity) or indirectly (such as chromatin unpacking, facilitating nuclease attack). Nonetheless, taking these observations as a whole, we suggest that the combined action of NE and MPO may have a dual role, firstly in intracellularly initiating NET formation and secondly in participating in NET degradation in the extracellular milieu (i.e., blood), which would point to a potential NET autocatabolism.

Conversely, other NET constituents may be involved in protecting DNA from nucleases such as the antimicrobial peptide LL-37 [72]. Alternatively, thrombin may bind to NET, thus conferring mutual protection against nuclease and protease degradation [73], illustrating the complex interplay between coagulation and NET formation [74].

Note, we found low but significant levels of both enzymes in the circulation of healthy individuals. We believe that the homeostasis of both enzymes and of nucleases such as DNase1L3 or DNase1 is critical in controlling NET formation on the one hand and cirDNA plasma concentration on the other. We speculate that genetic or epigenetic alterations impacting the expression of these entities may mitigate or exacerbate both the damage-associated molecular patterns (DAMPs) of cirDNA [3, 75] and NET formation, leading to chronic or acute disorders.

### NET association with several disorders

NET formation is an efficient, innate response strategy for counteracting intrusive microorganisms. However, exacerbated NET formation in the host can be detrimental, on account of the toxicity of its exposed compounds to endothelial cells and to parenchymal tissue [56], whose pathological consequences can include thrombosis and fibrinolysis disorders. For instance, NET components (DNA, histones, and granule proteins such as MPO or NE) may trigger an inflammatory process. Thus, the release of NET byproducts as a result of dysregulated NET formation, therefore, can be implicated in both autoimmune and non-autoimmune diseases [76–78].

Intense investigation has been done on the deleterious role of NET in autoimmune diseases, particularly in the pathogenesis of systemic lupus erythematosus (SLE). The dysregulation of NET formation has been repeatedly demonstrated in SLE [79, 80]; patients with SLE, notably, have increased levels of NET in their tissues and circulation. Thus, NET lead to the exposition of many autoantigens within the extracellular space, in particular double-strand DNA, and MPO. As a result, autoimmune complexes containing nucleic acids associated with various proteins (including antibodies, the chromatin-associated protein HMGB1, the antimicrobial peptide LL39, and ribonucleoproteins) are found in the sera of SLE patients [80]. Evidence has also been presented that the ability to degrade NET is impaired in patients with SLE [81], leading to a continuous release of interferon. Since this key cytokine in the SLE pathophysiology is also responsible for sensitizing neutrophils to NET formation, the overall result is the establishment of an amplifier loop which (with other factors) enables the autoimmune reaction to be sustained, and the disease to progress. Given all of the above, NET byproducts are now used as biomarkers for SLE clinical diagnostics [82]. In our study, we found a strong association of cirDNA with NE and MPO, as well as a negative correlation of MNR with NET-related biomarkers in the plasma of SLE patients [83]. These results are similar to those found in the supernatant of ex vivo activated neutrophils (Fig. 3), suggesting NET involvement in the pathogenesis of SLE.

Several recent reports have also described the involvement of NET formation in cancer progression [84–88]. Some authors even postulate that NET are a seedbed for metastasis [88]. We recently demonstrated the association of MPO and NE concentrations with the amounts of cirDNA and anti-cardiolipin auto-antibodies in a large cohort (*N* = 217) of newly diagnosed metastatic colorectal cancer (mCRC) patients [26]. In this current study, we clearly confirmed this observation in a small group of mCRC patients, in whom we found a statistically significant higher level of NE, MPO, and cirDNA plasma concentration, as compared to healthy individuals.

We are among the first to indicate that NET and NET byproducts play a key role in COVID-19 pathogenesis [25, 78, 89]. In this regard, notably, we have shown strong analogies between the multiple biological and pathological features, risk factors, and general clinical conditions observed in COVID-19 with several other disorders in which the uncontrolled formation of NET and their byproducts is implicated [25, 78, 89]. We also hypothesized that just such a deregulated NET formation is critically involved in the progression of COVID-19 under an amplifier loop, in a massive, uncontrolled inflammatory process [78]. Zuo et al. [90] were the first to experimentally show that cirDNA as well as NET protein byproducts are associated with COVID-19 and might be potential COVID-19 markers. Like a number of other authors, we have here confirmed that NE, MPO, and cirDNA concentrations in the plasma of patients with severe COVID-19 are significantly higher than in a large cohort of healthy individuals. We are now exploring the clinical validation of NET biomarkers in an ongoing clinical study which includes 160 patients.

Altogether, we found a significant increase in both NET proteins and cirDNA concentration in the plasma of SLE, mCRC, and COVID-19 patients (Fig. 7). Both NE and MPO with cirDNA markers (DII, cir-nDNA) showed strong positive correlations in patients with these three inflammatory diseases. These results confirm our in vitro and ex vivo experiments, adding further weight to the case for NET being one of the sources of cirDNA. We also found a constant and strong negative correlation of MNR with NET biomarkers, suggesting that the release and degradation rate of mitochondrial DNA could be associated with NET production, resulting in a decrease of the MNR in NET-associated disorders.

The topological features and extracellular mechanisms of the degradation of cir-mtDNA appear to be different from those of cir-nDNA and are currently under intense investigation in our laboratory [25, 50]. To these observations must be added those of our current study, which is the first to demonstrate that the cirDNA size profile of subjects suffering from SLE, mCRC, and COVID-19 has features distinct from that of healthy individuals, notably a cirDNA fragmentation specifically induced by a high level of NET formation. Overall, the size profiles of COVID-19 and mCRC patients, and to a lesser extent SLE patients, demonstrate a subtle but significant shift towards short DNA fragments, all of which indicates increased fragmentation of cirDNA. Note, the study is somewhat limited by the number of subjects, especially as compared to a clinical assay. The number of subjects nonetheless allowed us to observe significant statistical differences between groups and to draw preliminary observations. These will have to be confirmed with a larger number of patients to obtain more solid conclusions as to the impact of pathological NET dysregulation. Our study on quantitative values, association assays, and cirDNA size profile showed significant differences in patients versus HI, but remains exploratory and must be confirmed in larger patient cohorts. Overall, this exploratory work on the plasma of patients with diseases implying high levels of both NET formation and cirDNA (as compared to the plasma of HI) suggests (i) a specific and high association of cirDNA amount with NET protein markers, and (ii) a modification of the fragmentome of cirDNA from these patients, in whom NET may therefore be a significant source of cirDNA. This may imply that the proportion of NET as a cirDNA source is higher in these diseases, or at least that in these patients there is a difference in the respective proportion of the various mechanisms of cirDNA release mentioned above.

## Conclusions

The detection of cirDNA in blood is characteristically associated with the protective effect of mononucleosomes, which determines its fragmentation. The co-occurring observation that mononucleosomes clearly accumulate in cfDNA extracts following incubation of gHMW DNA in serum, or in plasma cirDNA extracts from mice or human subjects, in combination with the observation that cultured activated neutrophil produced NET and high molecular weight DNA that are further degraded mainly to mononucleosomes when incubated in cell culture medium where apoptosis cannot occur, strongly suggests that in a physiological setting NET may produce cirDNA as a byproduct, independent of apoptosis. The contribution of this process to cirDNA levels compared to other known mechanisms of release, such as apoptosis or necrosis, needs to be evaluated in healthy individuals and may have diagnostic potential with regard to diseases with NETopathies, such as some inflammatory diseases. Our work thus describes the previously unknown mechanistic bridge linking NET and cirDNA and establishes a new paradigm of the mechanism of cirDNA release in both normal and pathological conditions. In addition, our observations establish a link between immune response and cirDNA, thus enlarging the scope of the diagnostic potential offered by cirDNA, and giving a better understanding of the pathogenesis of inflammatory diseases.

## Supplementary Information

**Additional file 1: Fig. S1.** Analysis of the HMW DNA (chromatin) from cell lysate before degradation. (A) Q-PCR analysis of DNA concentration and DNA integrity index (DII). DNA of chromatin was amplified with primers targeting SRY gene (short F1/R1 73 bp and long F8/R1 246 bp) and primers targeting KRAS gene (short B1/B2 63 bp and long A1/B2 300 bp) (B) Capillary electrophoresis analysis of DNA size profile. **Fig. S2.** Kinetics of NET degradation in blood fluids. Kinetics of DII (DNA integrity index) following the degradation of NET (supernatant of PMA activated neutrophils) by incubation in serum/plasma up to 24 h: before incubation (S0) and after incubation at 37°C in plasma for 24 h or in serum for 5 min/10 min/20 min/30 min/2 h/8 h/24 h. Data are represented as mean ± SD. **Fig. S3.** Kinetics of NET production *in vitro*. The kinetics of cfDNA, as determined by Q-PCR, in the supernatant of control non-stimulated (gray) and stimulated by 1 mg/ml LPS (blue) or 100 nM PMA (red) human neutrophils from a healthy donor following incubation up to 5 h at 37°C in cell culture: (A) DII (DNA integrity index) of nuclear cfDNA; (B) DII (DNA integrity index) of mitochondrial cfDNA. Data are represented as mean ± SD. (C) Correlation matrix of MPO and NE concentrations (ng/mL plasma) with cirDNA parameters in supernatant of the same control non-stimulated and stimulated (LPS and PMA) neutrophils. The heatmap manifests the strength of the relationship by Pearson’s correlation analysis (red: positive correlation; blue: negative correlation). NE: neutrophil elastase; MPO: myeloperoxidase; cir-nDNA: total cell-free DNA of nuclear origin; nDNA long: long (>300 bp) cell-free DNA of nuclear origin; nDNA DII: DNA integrity index of nuclear DNA; cir-mtDNA: circulating cell-free DNA of mitochondrial origin; mtDNA long: long (>310 bp) cell-free DNA of mitochondrial origin; mtDNA DII: DNA integrity index of mitochondrial DNA; MNR: ratio of mitochondrial to nuclear DNA concentrations. **Fig. S4.** Representation of the crystal structure of the nucleosome core particle, chromatosome, and chromatosome with a flexible DNA chain, on the cfDNA fragment size profile of the thirteen healthy subjects, as determined by sWGS. Chromatosome with 167-bp DNA fragment is the most present cfDNA associated structure, while being of low frequency (~2%). The nucleosome core particle devoid of H1 containing 147 - 160 bp is the second most present structure (1.1% - 1.2%). Arrows on a nucleosome structure indicate the minor groove DNA sites subject to DNase attacks, explaining the ~10 bp periodic sub-peaks in size profile revealing nicks on the nucleosome-associated DNA. Images of the crystal structure of chromatosome and nucleosome at 3.5 angstrom resolution, from the NIPDB data bank (4QLC and 5ONW, respectively); NIPDB: http://npidb.belozersky.msu.ru/complex/clist.html. **Fig. S5.** Fragmentomics analysis of plasma cirDNA of WT, NE KO and AAT KO mice. (A) Values of several fragmentomic parameters of cirDNA calculated from sWGS data (DII). (B) P values for the cirDNA fragmentomics parameters when comparing the groups of mice. **Table S1. **Summary statistics of sWGS data. **Table S2.** Analysis of DNA fractions corresponding to mononucleosome (mono-N), dinucleosome (di-N) and trinucleosome (tri-N) before (S0) and after (S 5 min, S2H, S24H, P24H) degradation of gHMW DNA in blood fluids, calculated from sWGS data.

**Additional file 2. **Additional comments or details on the Results section.

## Data Availability

Raw sequencing reads for in vitro experiments and mice are available from Zenodo: https://doi.org/10.5281/zenodo.6949112 [39]. Patient raw sequencing reads are available from the EGA at EGAS00001006759 (https://ega-archive.org/studies/EGAS00001006759) [91]

## Abbreviations

NET: Neutrophil extracellular traps
gHMW DNA: Genomic high molecular weight DNA
NE: Neutrophil elastase
MPO: Myeloperoxidase
mono-N: Mononucleosome
di-N: Dinucleosome
tri-n: Trinucleosome
cirDNA: Circulating DNA
cir-nDNA: Circulating DNA of nuclear origin
cir-mtDNA: Circulating DNA of mitochondrial origin
LPS: Lipopolysaccharides
PMA: Phorbol 12-myristate 13-acetate
qPCR: Quantitative polymerase chain reaction
sWGS: Shallow whole genome sequencing
SLE: Systemic lupus erythematosus
mCRC: Metastatic colorectal cancer
HI: Healthy individuals
