# Supplementary material for: Neutrophil extracellular traps have auto-catabolic activity and produce mononucleosome-associated circulating DNA
Source: Genome Med. 2022 Nov 28;14:135. doi: 10.1186/s13073-022-01125-8 (PMC9702877; doi:10.1186/s13073-022-01125-8)
Supplement: Supplementary file 1 — Additional file 1: Fig. S1. Analysis of the HMW DNA (chromatin) from cell lysate before degradation. (A) Q-PCR analysis of DNA concentration and DNA integrity index (DII). DNA of chromatin was amplified with primers targeting SRY gene (short F1/R1 73 bp and long F8/R1 246 bp) and primers targeting KRAS gene (short B1/B2 63 bp and long A1/B2 300 bp) (B) Capillary electrophoresis analysis of DNA size profile. Fig. S2. Kinetics of NET degradation in blood fluids. Kinetics of DII (DNA integrity index) following the degradation of NET (supernatant of PMA activated neutrophils) by incubation in serum/plasma up to 24 h: before incubation (S0) and after incubation at 37°C in plasma for 24 h or in serum for 5 min/10 min/20 min/30 min/2 h/8 h/24 h. Data are represented as mean ± SD. Fig. S3. Kinetics of NET production in vitro. The kinetics of cfDNA, as determined by Q-PCR, in the supernatant of control non-stimulated (gray) and stimulated by 1 mg/ml LPS (blue) or 100 nM PMA (red) human neutrophils from a healthy donor following incubation up to 5 h at 37°C in cell culture: (A) DII (DNA integrity index) of nuclear cfDNA; (B) DII (DNA integrity index) of mitochondrial cfDNA. Data are represented as mean ± SD. (C) Correlation matrix of MPO and NE concentrations (ng/mL plasma) with cirDNA parameters in supernatant of the same control non-stimulated and stimulated (LPS and PMA) neutrophils. The heatmap manifests the strength of the relationship by Pearson’s correlation analysis (red: positive correlation; blue: negative correlation). NE: neutrophil elastase; MPO: myeloperoxidase; cir-nDNA: total cell-free DNA of nuclear origin; nDNA long: long (>300 bp) cell-free DNA of nuclear origin; nDNA DII: DNA integrity index of nuclear DNA; cir-mtDNA: circulating cell-free DNA of mitochondrial origin; mtDNA long: long (>310 bp) cell-free DNA of mitochondrial origin; mtDNA DII: DNA integrity index of mitochondrial DNA; MNR: ratio of mitochondrial to nuclear DNA concentrations. Fig. S4. Repr [file 13073_2022_1125_MOESM1_ESM.docx]

**Figure S1**

**A**

**B**


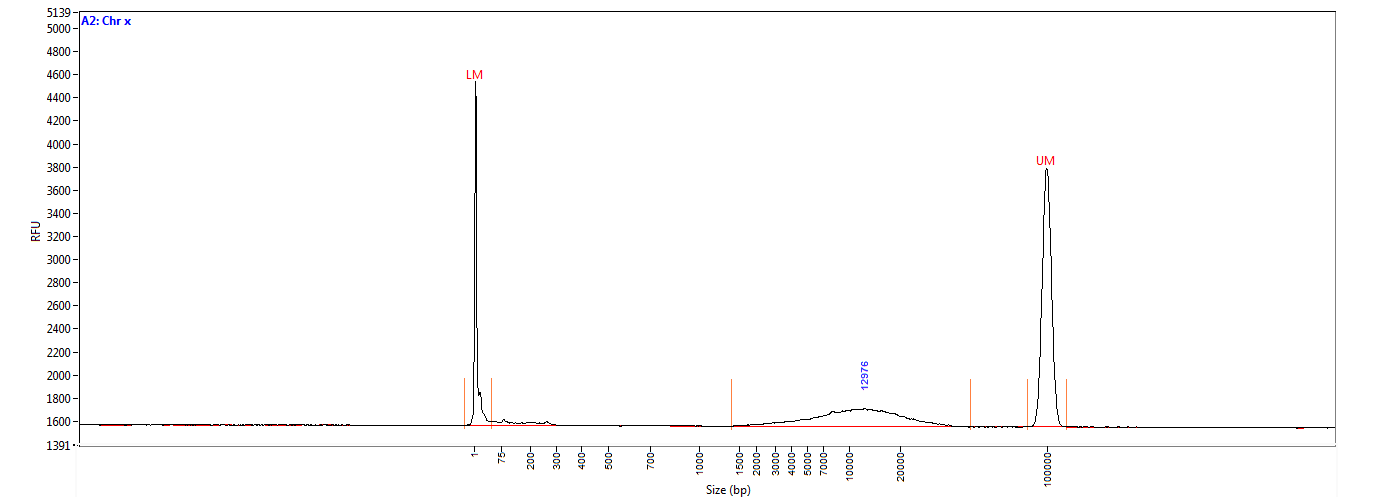


**Conclusion:** cellular extract contains non-degraded chromatin that could be used for the study of degradation of long-sized gDNA (as gHMW DNA) in blood.

**Figure S1: Analysis of the HMW DNA (chromatin) from cell lysate before degradation.** (A) Q-PCR analysis of DNA concentration and DNA integrity index (DII). DNA of chromatin was amplified with primers targeting SRY gene (short F1/R1 73 bp and long F8/R1 246 bp) and primers targeting KRAS gene (short B1/B2 63 bp and long A1/B2 300 bp) (B) Capillary electrophoresis analysis of DNA size profile.

**Figure S2**

**Figure S2: Kinetics of NET degradation in blood fluids.** Kinetics of DII (DNA integrity index) following the degradation of NETs (supernatant of PMA activated neutrophils) by incubation in serum/plasma up to 24 hours: before incubation (S0) and after incubation at 37°C in plasma for 24 hours or in serum for 5 min/10 min/20 min/30 min/2 hours/8 hours/24 hours. Data are represented as mean ± SD.

**Figure S3**


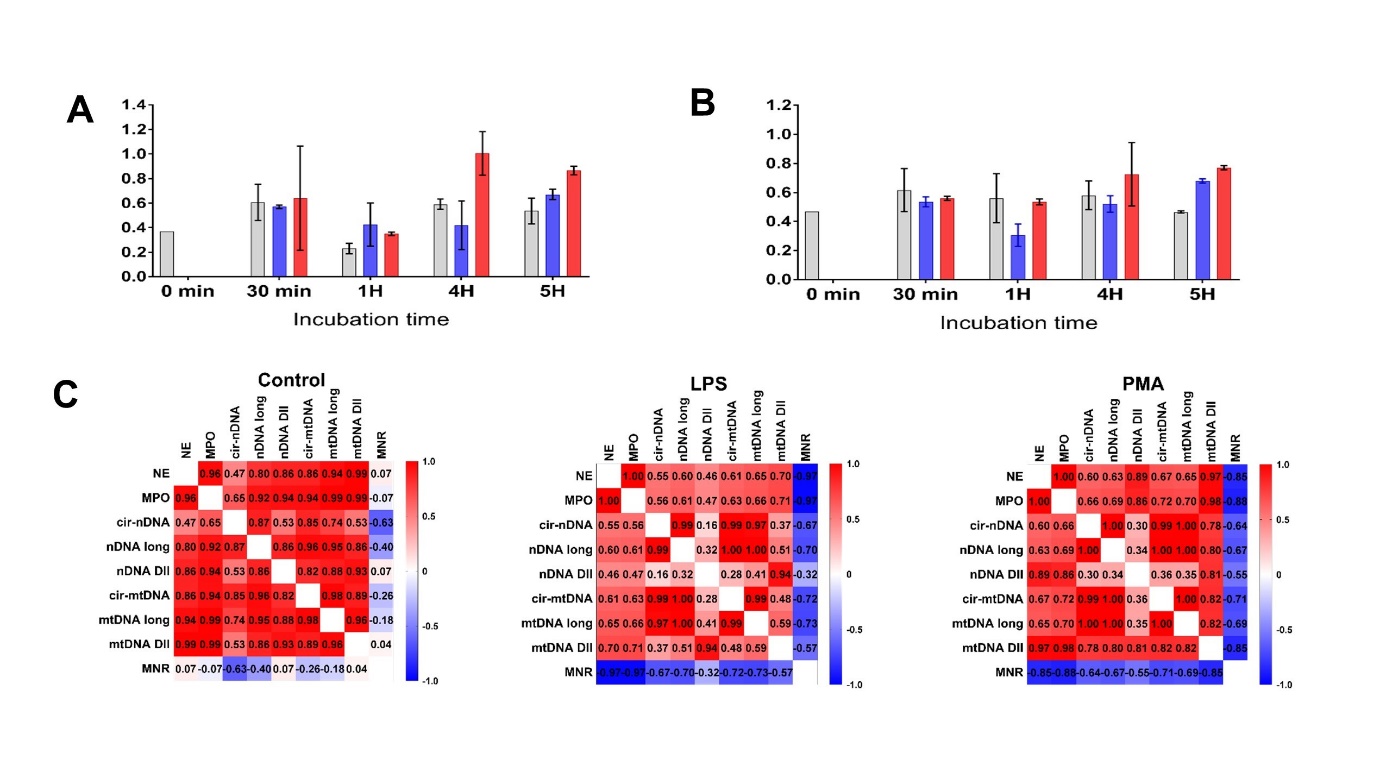


**Figure S3: Kinetics of NET production *in vitro*.** The kinetics of cfDNA, as determined by Q-PCR, in the supernatant of control non-stimulated (grey) and stimulated by 1 mg/ml LPS (blue) or 100 nM PMA (red) human neutrophils from a healthy donor following incubation up to 5 hours at 37°C in cell culture: (A) DII (DNA integrity index) of nuclear cfDNA; (B) DII (DNA integrity index) of mitochondrial cfDNA. Data are represented as mean ± SD. (C) Correlation matrix of MPO and NE concentrations (ng/mL plasma) with cirDNA parameters in supernatant of the same control non-stimulated and stimulated (LPS and PMA) neutrophils. The heatmap manifests the strength of the relationship by Pearson’s correlation analysis (red: positive correlation; blue: negative correlation). NE: neutrophil elastase; MPO: myeloperoxidase; cir-nDNA: total cell-free DNA of nuclear origin; nDNA long: long (>300 bp) cell-free DNA of nuclear origin; nDNA DII: DNA integrity index of nuclear DNA; cir-mtDNA: circulating cell-free DNA of mitochondrial origin; mtDNA long: long (>310 bp) cell-free DNA of mitochondrial origin; mtDNA DII: DNA integrity index of mitochondrial DNA; MNR: ratio of mitochondrial to nuclear DNA concentrations.

**Figure S4**


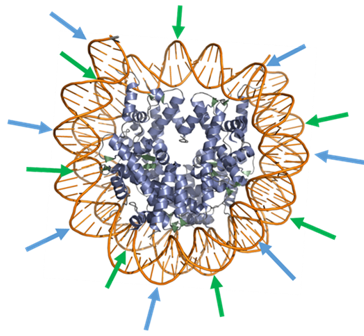

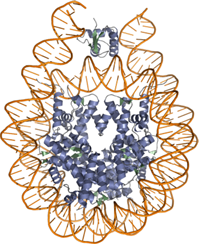

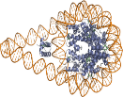


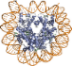


**Nucleosome**

**core particle**

**(145 bp)**

**DNase 1 nicks on nucleosome**

**core particle DNA**

**nicks on one turn**

**nicks on the other**

**(7 + 7 potential nick sites)**

**Chromatosome**

**with linker histone H1**

**(167 bp)**

**Chromatosome**

**with linker histone H1**

**(197 bp)**

**Figure S4: Representation of the crystal structure of the nucleosome core particle, chromatosome, and chromatosome with a flexible DNA chain, on the cfDNA fragment size profile of the thirteen healthy subjects, as determined by sWGS.** Chromatosome with 167-bp DNA fragment is the most present cfDNA associated structure, while being of low frequency (~2%). The nucleosome core particle devoid of H1 containing 147 - 160 bp is the second most present structure (1.1% - 1.2%). Arrows on a nucleosome structure indicate the minor groove DNA sites subject to DNase attacks, explaining the ~10 bp periodic sub-peaks in size profile revealing nicks on the nucleosome-associated DNA. Images of the crystal structure of chromatosome and nucleosome at 3.5 angstrom resolution, from the NIPDB data bank (4QLC and 5ONW, respectively); NIPDB: <http://npidb.belozersky.msu.ru/complex/clist.html>

**Figure S5**

**A**

| **Parameter** | **WT** | | | **NE KO** | | | **AAT KO** | | |
| --- | --- | --- | --- | --- | --- | --- | --- | --- | --- |
|  | **MEAN** | **MEDIAN** | **SD** | **MEAN** | **MEDIAN** | **SD** | **MEAN** | **MEDIAN** | **SD** |
| **% 168 bp/Fraction 168-260 bp** | 0,05 | 0,05 | 0,01 | 0,04 | 0,04 | 0,01 | 0,05 | 0,05 | 0,00 |
| **% 168 bp/% 351 bp** | 27,6 | 30,6 | 8,1 | 14,0 | 14,0 | 3,0 | 21,1 | 22,3 | 4,0 |
| **% 168 bp/Fraction 260-450 bp** | 0,28 | 0,30 | 0,08 | 0,14 | 0,13 | 0,03 | 0,21 | 0,23 | 0,04 |
| **% 168 bp/(%152-%142)** | 20,7 | 8,5 | 45,6 | 7,9 | 7,5 | 0,9 | 8,5 | 8,2 | 1,1 |
| **% 152 - % 142** | 0,11% | 0,09% | 0,11% | 0,21% | 0,22% | 0,03% | 0,21% | 0,22% | 0,03% |
| **% at the subpeak 142-144** | 1,24% | 1,16% | 0,25% | 0,83% | 0,80% | 0,06% | 0,99% | 0,98% | 0,12% |
| **% at the subpeak 152-154** | 1,36% | 1,37% | 0,16% | 1,03% | 1,03% | 0,08% | 1,20% | 1,19% | 0,11% |
| **fraction <167 bp** | 58% | 56% | 7% | 41% | 46% | 18% | 53% | 53% | 4% |
| **Fraction 167 – 260 bp** | 34% | 34% | 5% | 44% | 38% | 17% | 37% | 36% | 2% |
| **Fraction 260 – 450 bp** | 6,7% | 6,1% | 1,9% | 12% | 12% | 2% | 8,7% | 8,2% | 1,6% |
| **Fraction 450 – 650 bp** | 1,26% | 1,04% | 0,45% | 2,7% | 2,9% | 0,6% | 1,58% | 1,38% | 0,50% |
| **% at 167 bp** | 1,73% | 1,72% | 0,13% | 1,60% | 1,64% | 0,09% | 1,77% | 1,81% | 0,09% |

**B**

| **Parameter** | **P values** | | |
| --- | --- | --- | --- |
|  | **WT vs NE KO** | **WT vs AAT KO** | **NE KO vs AAT KO** |
| **% 168 bp/ Fraction 168-260 bp** | 0,0137 | 0,1621 | **0,0146** |
| **% 168 bp/% 351 bp** | **0,0013** | **0,0480** | **0,0023** |
| **% 168 bp/Fraction 260-450 bp** | **0,0014** | **0,0356** | **0,0016** |
| **% 168 bp/(%152-%142)** | 0,4722 | 0,4042 | 0,2932 |
| **% 152 - % 142** | **0,0226** | **0,0040** | 0,2331 |
| **% at the subpeak 142-144** | **0,0013** | **0,0117** | **0,0180** |
| **% at the subpeak 152-154** | **0,0008** | **0,0469** | **0,0064** |
| **Fraction <167 bp** | **0,0363** | 0,0575 | 0,0617 |
| **Fraction 167 – 260 bp** | 0,1493 | 0,1080 | 0,1910 |
| **Fraction 260 – 450 bp** | **0,0001** | **0,0255** | **0,0039** |
| **Fraction 450 – 650 bp** | **0,0002** | 0,1882 | **0,0010** |
| **% at 167 bp** | 0,0650 | 0,4156 | **0,0033** |

**Figure S5: Fragmentomics analysis of plasma cirDNA of WT, NE KO and AAT KO mice.** (A) Values of several fragmentomic parameters of cirDNA calculated from sWGS data (DII). (B) P values for the cirDNA fragmentomics parameters when comparing the groups of mice.

**Table S1. Summary statistics of sWGS data**

**Table S2.** Analysis of DNA fractions corresponding to mononucleosome (mono-N), dinucleosome (di-N) and trinucleosome (tri-N) before (S0) and after (S 5 min, S2H, S24H, P24H) degradation of gHMW DNA in blood fluids, calculated from sWGS data.

| **Fraction** | **mono-N** | **di-N** | **tri-N** |
| --- | --- | --- | --- |
| **Sample** | **41-260 bp** | **261-460 bp** | **461-700 bp** |
| **S0** | 0% | 0% | 0% |
| **S 5 min** | 57.4% | 29.3% | 13.1% |
| **S 2H** | 64.8% | 28.1% | 7.1% |
| **S 24H** | 81.3% | 17.6% | 1.1% |
| **P 24H** | 66.1% | 21.2% | 12.4% |
