# Supplementary material for: Neutrophil extracellular traps have auto-catabolic activity and produce mononucleosome-associated circulating DNA
Source: Genome Med. 2022 Nov 28;14:135. doi: 10.1186/s13073-022-01125-8 (PMC9702877; doi:10.1186/s13073-022-01125-8)
Supplement: Supplementary file 2 — Additional file 2. Additional comments or details on the Results section. [file 13073_2022_1125_MOESM2_ESM.docx]

**Additional file 2**

**Additional comments or details on the Results section**

### Kinetics of gHMW DNA degradation in blood fluids

The Q-PCR quantification of *SRY* DNA sequences showed no significant change of total or long DNA fragment concentration, or of the DNA integrity index (DII) in blood and plasma EDTA up to 24 hours (Fig. 2A). Thus, no or poor amount of fragments are below 250 bp in these two conditions. ). This may be explained by the presence of EDTA, which is known to be a strong inhibitor of nucleases [8]. Since there is no EDTA in serum, the numerous blood DNases are able to nick gHMW DNA. In contrast, the total DNA concentration decreased down to 18%, and the concentration of long DNA fragments decrease down to 13% of the initial concentration during the first 30 min of incubation of HMW DNA in serum. The concentration of long DNA fragments decreased faster than the concentration of total DNA, as illustrated by decrease of DII. This clearly suggested appearance of a significant fragmentation and preponderance of fragments of low size. We estimated the T_1/2_ of HMW DNA in serum as 18 min.

Shallow whole genome sequencing (sWGS) precisely determines DNA size profiles in the 30 – 1000 bp range. Using a resolution of 1 bp, in our study it revealed a remarkable homogeneity of size profile in its analysis of 13 healthy individuals (Fig. 2C). The cirDNA size profile showed the chromatin organization pattern of DNA fragment distribution with a main peak at 167 bp and another of lower frequency at 340 bp, corresponding to chromatosome/mononucleosome-associated (mono-N) DNA and dinucleosome-associated (di-N) DNA, respectively [29,34–36].

Control untreated gHMW DNA size profile showed a maximum of fragments around 50 bp, and a uniform pattern of slow decrease in fragment length from 50 down to 650 bp (Fig. 2B). the size profile of gHMW DNA before incubation that did not show any peaks. These fragments below ~1000 bp constituted uncharacterized waste DNA, and represent a tiny and negligible part of the total DNA quantity, as we demonstrated by capillary electrophoresis before degradation (Additional file 1: Fig. S1).

gHMW DNA incubation in serum showed a progressive degradation of DNA, including a decrease of the total DNA and long DNA fragment concentrations, as well as a decrease of DII. Degradation showed itself to be very rapid, given that we were able to observe the emergence of mono-N and di-N DNA fragments after only 5 min of incubation of gHMW DNA in serum. In contrast, our data revealed the absence or near-absence of gHMW DNA degradation in blood EDTA and plasma, and indicated no change in the fraction of mononucleosome-associated (mono-N) fragments (Fig. 2. , Additional file 1: Table 2S).

### Kinetics of ex vivo NET production

Neutrophils isolated by negative immune selection from the EDTA blood of a healthy donor were incubated in cell culture without stimulation (control) and with stimulation by LPS and PMA which are efficient stimulators of NET production *ex vivo* [37]. The supernatant of stimulated neutrophils was isolated by double centrifugation to guarantee the absence of cells and cell debris. NETs associated proteins such as NE and MPO were then quantified by ELISA, and the extracellular DNA was analyzed using Q-PCR, sWGS, and capillary electrophoresis.

This study allowed us to assess the kinetics of DNA production by NETs *ex vivo,* and its degradation in serum.

The DNA integrity index (DII) of nuclear (Additional file1: Fig. S4A) and mitochondrial DNA (Additional file1: Fig. S4B) did not demonstrate any consistent changes in stimulated or non-stimulated neutrophils. We observed a progressive decrease of mitochondrial to nuclear DNA ratio (MNR) in both stimulated and non-stimulated neutrophils was observed (Fig. 3C) which was more pronounced in PMA stimulated neutrophils given the sharp MNR increase rapidly after the start of stimulation (30min) (Fig. 3C).

The Pearson correlation study revealed strong positive correlations between DNA and NETs protein (NE, MPO) markers in control and stimulated neutrophils. The total cf-nDNA correlates positively with NETs in PMA (NE: r=0.60; MPO: r=0.66) or LPS (NE: r=0.55; MPO: r=0.56) stimulated neutrophils, as well as in non-stimulated neutrophils (NE: r=0.47; MPO: r=0.65). The correlations of the long cf-nDNA and cf-mtDNA fragments with NE/MPO were slightly higher than that of the total cf-nDNA in stimulated and non-stimulated neutrophils, suggesting that production of long fragment or cir-mtDNA might be correlated with NETs formation. Alternatively, the MNR negatively associated with NE and MPO in the supernatant of stimulated neutrophils that distinguished them from the control (non-stimulated) cells.

Since sWGS is technically limited to a maximum length of 1000 bp for DNA fragment analysis, we also assessed the kinetics of HMW DNA degradation by capillary electrophoresis, this being the only method of assessing fragment distribution profile in the 1K - 10K bp range. (It must be noted that this analysis method is less sensitive than sWGS.) Our data revealed that activated neutrophils produce HMW DNA fragments ranging in length from 1500 to 30 000 bp, with the average being 8653 bp (Fig. 3H). In order to visualize these NETs, we performed Hoechst staining of control and stimulated neutrophils. Due to the specific affinity of Hoechst staining for DNA, we were able to observe DNA strands around multilobed nuclei that correspond to activated neutrophils releasing NETs (Fig. 3G,H). These NET images are consistent with those already described by other researchers [48,49].

Note, the 10 bp periodicity which revealed the nucleosomal pattern was most pronounced at baseline (PMA0), then gradually disappeared with incubation time (Fig. 3E).

In our present study, we describe the accumulation of HMW DNA fragments and the increase of NETs markers in the supernatant of *ex vivo* activated neutrophils. The supernatants of these PMA stimulated neutrophils showed the simultaneous increase of extracellular DNA, NE and MPO, in contrast to control non-stimulated neutrophils. This would indicate the formation of these DNA fibers decorated with NETs-specific proteins, such as MPO and NE. Our correlation analysis confirms these results by revealing the significant correlations of the DNA markers (based on size and amount) with MPO and NE.

### Kinetics of in vitro NETS degradation in blood

Q-PCR quantification revealed the progressive decrease of the total DNA and the long DNA fragment concentrations (Fig. 4A), as well as the synchronous decrease of the DII (Additional file 1: Fig. S5).

Analysis of the same DNA extracts by capillary electrophoresis demonstrated the shortening and dynamic reduction of the 1 - 30 kb DNA fraction during incubation in serum (Fig. 4B). That observation agrees with the Q-PCR results. The analysis of DNA by capillary electrophoresis does not allow us to distinguish male and female DNA using the sequence of the Y chromosome and therefore to directly quantify their respective amount. However, given the 30-fold excess of DNA from NETs (0.36 ng/μL) compared to cirDNA from serum (0.012 ng/μL), we can assume that more than 95% of the DNA fragments detected by capillary electrophoresis originate from NETs.

The sWGS revealed, in the course of NETs degradation, a typical chromatin pattern with a most prominent peak corresponding to mono-N, a less prominent peak of di-N, and traces of tri-N (Fig. 4C). The strong depletion of long DNA fragments resulted in the formation of oligonucleosomes, making them the main substrate for DNase attacks, and leading to the subsequent accumulation of mono-N and di-N (after 2H incubation). This also led to the accumulation of short DNA fragments in the 40 - 160 bp range, indicating the dynamic action of serum nucleases on the internucleosomal DNA, the DNA linked to the histone H1, and the 14 DNA base pairs exposed at the surface of the nucleosome core particle at a ~10 bp periodicity [29,35,36]. Note, in plasma which had undergone incubation at 37°C for 24h, we also observed the emergence of a very small mono-N DNA fraction, indicating the imperfect inhibition of nucleases by EDTA. The most pronouced changes were detected for the 461 - 760 bp fraction (tri-N), which showed a 10-fold decrease, while fragments corresponding to the mono-N fraction increased by more than half during incubation in serum.

The NETs production was associated with the accumulation of di-N and tri-N DNA and, thus, with a decrease of the relative fraction of short fragments (mono-N). Conversely, in the course of the degradation of NET DNA in serum we observed an inversion of all those proportions, with the progressive disappearance of di-N and tri-N associated DNA fragments, and the accumulation of mono-N DNA fragments (<121 bp) (Fig. 4C). These results confirm our hypothesis that, when expelled to the extracellular milieu (in this case, the serum), the long fragments of DNA originating from NETs are predominantly degraded to mononucleosomes, given that (of all NETs byproducts) these constitute the most stabilized structure with which cirDNA can associate.

### gHMW DNA degradation in presence of NE and MPO

**Raw data values:**

**Overall observations:**

1. Addition of NE and/or MPO in plasma or in serum greatly improved HMW DNA degradation (Fig. 5A). For instance, only ~1% of DNA remain in serum following 2H or 8H incubation with both NE and MPO, as compared to ~41% and 27% without NE and MPO after 2H and 8H incubation, respectively.

In term of degradation activity NE+MPO in serum 4.1 vs 2.4 ng/mL/min. in 2 hours

NE+MPO in serum 1.03 vs 0.78 ng/mL/min. in 8 hours

For instance, addition of NE plus MPO in serum enhanced by nearly the double (4.1 vs 2.4 ng/mL/min.) of the degradation activity in the first 2 hours of incubation.

1. NE or MPO both improve HMW DNA degradation in plasma following 2H (40% and 68%, respectively) and 8H (35% and 66%, respectively) incubation. 19% and 16% of DNA remain in plasma following 2H or 8H incubation with both NE and MPO, as compared to ~95% and 85% without NE and MPO after 2H and 8H incubation, respectively.
2. NE and/or MPO addition improved nuclease activity by 30% and 36% (2H) or 20% and 22% (8H), respectively.
3. MPO showed approximately twice as much effect on DNA degradation than NE.
4. NE and MPO may act separately to improve DNA degradation
5. There is only a slight improvement when combining NE and MPO.
6. Since SRY sequence was targeted by q-PCR, there was no possibility of bias potentially deriving from ndogenous DNA from cirDNA initially contained in plasma or serum

We also observed a decrease in the amount of gHMW DNA detected in plasma following the addition of NE and/or MPO. The presence of NE or MPO during 2h incubation of gHMW DNA in plasma led to a 2-fold and 5-fold decrease, respectively, of total DNA concentration, as compared to 2H control incubation (257 ng/ml and 93 ng/ml vs 475 ng/ml) (Fig. 5A). The 8H incubation in plasma showed similar results to the 2h incubation. The simultaneous addition of both NE and MPO showed the same variations, in that 8h incubation led to a 5-fold decrease of the total DNA concentration, as compared to control incubation (82 ng/ml vs 424 ng/ml) (Fig. 5A).

From the data obtained in Fig 5A, we could estimate the capacity of NE and/or MPO to effect HMW DNA degradation rate. The estimated **degradation rate within** the first two hours of incubation is 3- and 4-fold higher than that observed during the 2-8 hours incubation period for controls and NE/MPO containing serum. For instance, the degradation rate estimated during 2 and 8 hours of incubation in serum containing the combination of NE and MPO is 4.1 and 1.03 ng/mL/minutes, respectively. The degradation rate within the first two hours of incubation is 3- and 4-fold higher than that observed during the 2-8 hours incubation period for controls and NE/MPO containing plasma.


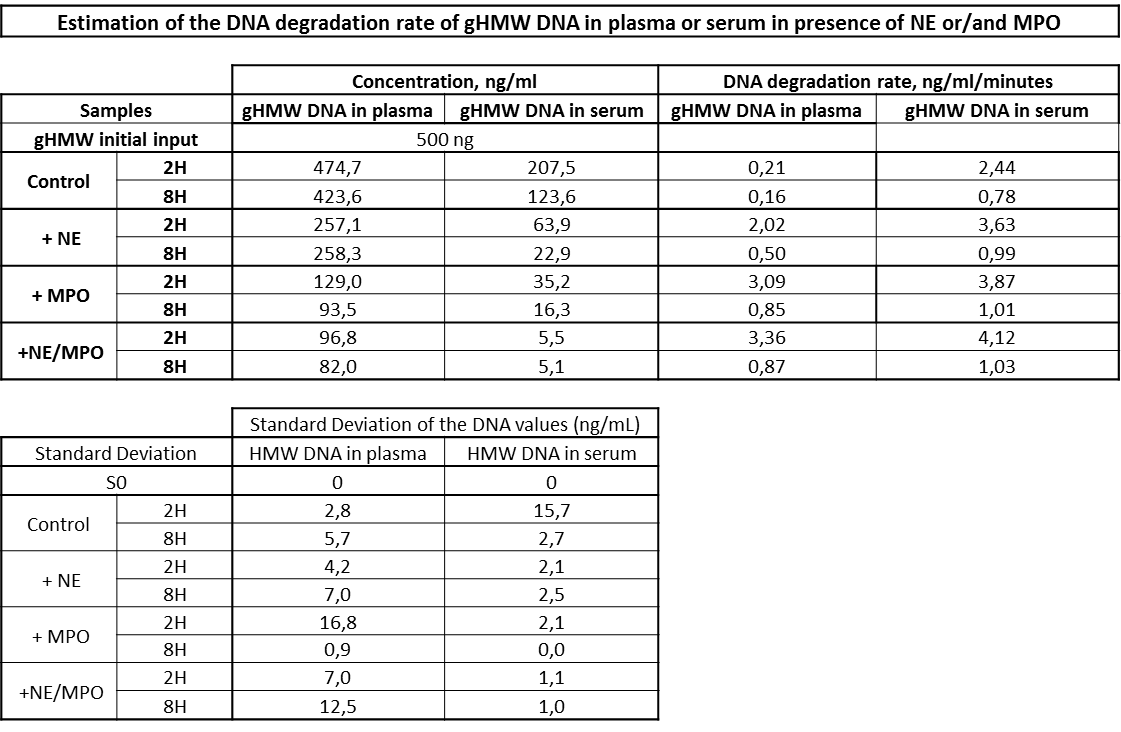


Overall, ata suggest that: (i), addition of NE and/or MPO in plasma or in serum greatly improved HMW DNA degradation (Fig. 5A); (ii), NE and MPO independently improved HMW DNA degradation; (iii), NE and/or MPO greatly improved DNA degradation in presence of high nuclease activity; (iv) MPO showed approximately twice as much increase of DNA degradation than NE; and (v), NE and MPO may act separately to improve DNA degradation, while there is only a slight improvement when combining NE and MPO.. .

As for the evolution of the proportions of the fragment range population, by comparison with incubation in serum alone, incubation with MPO and NE alone showed a sharp increase up to 2H and plateauing up to 8H at 34%, 25%, 14% increase of the mono-N (120-280 bp), di-N (281-450 bp) and tri-N (451-700 bp) DNA proportions, respectively (Fig. 5C). Incubation with combined MPO and NE combined showed the same curve shape but exhibiting higher, similar and lower proportion in the course of the 8H time period plateauing at 48%, 25% and 8%, respectively. (Fig. 5C).

### In vivo study on the role of NE in cirDNA fragmentome

The average fragment length of NE KO cirDNA was significantly higher than that of WT cirDNA (193 bp vs 179 bp, p<0.01) or AAT KO cirDNA (193 bp vs 181 bp, p<0.01). The di-N population of the NE KO mice plasma peaked at 355 bp, and was more prominent than the same population of the WT or AAT KO mice (Fig. 6A).

We also used fragmentomic analysis to compare several parameters in these 3 groups of mice, which clearly distinguished the WT and AAT KO mice from the NE KO mice (Additional file 1: Fig. S2). For instance, the di-N and tri-N fractions ranging from 260 - 450 bp and 460 - 650 bp respectively, were both ~2-fold higher in NE KO mice than in the other two groups (P< 0.005). In addition, the shoulder (142 - 152 bp) previously described as a fragmentomic marker [29,35,39] was more than 2-fold more pronounced in AAT KO and NE KO mice than in WT mice (P< 0.05).

The absence of NE (in NE KO mice) was associated with the increase of the di-N associated cirDNA fragments and a significant increase of the average fragment size, which would indicate impaired DNA fragmentation, as compared to WT mice. The cirDNA fragment size profile of WT and AAT KO mice were visually similar, despite the increased activity of NE in the AAT KO mice. Capillary electrophoresis analysis revealed a decrease in the 1k - 10k bp cirDNA fragment fraction in AAT KO mice,

### NETs markers and cirDNA association in plasma in several disorders.

In order to study the association of cirDNA and other NETs markers in different disorders known to involve the production of both NETs and cirDNA, we analyzed the plasma samples from 11 systemic lupus erythematosus (SLE) patients, 10 metastatic colorectal cancer (mCRC) patients, 28 COVID-19 patients, and 114 healthy individuals (HI). We performed a quantification of NETs biomarkers and an analysis of their correlation, as well as a fragmentomic analysis of the cirDNA size profiles of the SLE, mCRC and COVID-19 patients and HI.

***Conclusion:***

In that context, we here report *in vitro*, *ex vivo* and *in vivo* validation of a new paradigm in which NETs are shown to produce stabilized circulating mononucleosomes under a specific autocatabolic mechanism and, as such, to be a potential source of cirDNA.
